# A Precision Engineered Interleukin-2 for Bolstering CD8^+^ T- and NK-cell Activity without Eosinophilia and Vascular Leak Syndrome in Nonhuman Primates

**DOI:** 10.1158/2767-9764.CRC-24-0278

**Published:** 2024-10-25

**Authors:** Lina Ma, Nicole V. Acuff, Ingrid B. Joseph, Jerod L. Ptacin, Carolina E. Caffaro, Kristine M. San Jose, Hans R. Aerni, Roberto Carrio, Anthony M. Byers, Rob W. Herman, Yelena Pavlova, Michael J. Pena, David B. Chen, Christen Buetz, Taylor K. Ismaili, Helene V. Pham, Margot Cucchetti, Ingrid Sassoon, Lilia K. Koriazova, Joseph A. Leveque, Laura K. Shawver, Jill M. Mooney, Marcos E. Milla

**Affiliations:** 1 Synthorx, Inc., A Sanofi Company, La Jolla, California.; 2 Sanofi, Orlando, Florida.; 3 Sanofi, Vitry-sur-Seine, France.

## Abstract

We have created a precisely pegylated IL-2 [SAR-444245 (SAR’245) or pegenzileukin, previously THOR-707] designed for proliferation of target CD8^+^ T and NK cells for anticancer activity, with minimal expansion of anti-target regulatory CD4^+^ T cells (Treg) that counter their action, or eosinophils that trigger vascular leak syndrome (VLS). We performed *in vivo* studies in nonhuman primates (NHP) to monitor the safety of SAR’245, pharmacokinetic profile, and pharmacodynamic parameters including expansion of peripheral CD8^+^ T and NK cells, and effects on Tregs and eosinophils. Studies included multiple ascending dosing and repeat dosing with different regimens (QW, Q2W, Q3W and Q4W). We also conducted *ex vivo* studies using human primary cells to further evaluate SAR’245 stimulation of target cells alone and in combination with programmed cell-death 1 (PD-1) checkpoint inhibitors. The pharmacokinetic profile of SAR’245 in NHP demonstrated dose-proportional exposure that was comparable with redosing. It elicited expansion of peripheral CD8^+^ T and NK cells that was comparable with each dose and with multiple dosing regimens. Once-weekly dosing showed no significant adverse effects, including no hallmark signs of VLS at dosing levels up to 1 mg/kg. *Ex vivo*, SAR’245 enhanced T-cell receptor responses alone and in combination with PD-1 inhibitors without inducing cytokines associated with cytokine release syndrome or VLS. Results support the clinical development of SAR’245 as a drug candidate for the treatment of solid tumors, alone or in combination with PD-1 inhibitory agents.

**Significance:**

SAR-444245 (SAR’245, pegenzileukin) is an extended half-life IL-2 that targets effector CD8^+^ T and NK cells, with little effect on regulatory T cells. We show that in the nonhuman primate model that closely approximates human immune function and response to IL-2, SAR’245 selectively activates CD8^+^ T and NK effectors without significant serious side effects (vascular leak syndrome or cytokine release syndrome), suggesting its potential for the treatment of solid tumors in humans.

## Introduction

IL-2 mediates many important immunologic functions, including the induction of lymphocyte proliferation and survival as well as upregulation of effector T (Teff) and NK cytotoxic cells activity. Aldesleukin, or high-dose (37 μg/kg) recombinant human IL-2 (rhIL-2), monotherapy has been shown to effect durable and, in some cases, complete responses in patients with metastatic renal cell carcinoma (RCC) and metastatic melanoma, but with objective responses generally observed for lesser than 20% of patients in clinical trials (1, 2). However, several drawbacks intrinsic to IL-2 limit its efficacy and clinical use at the approved dose level. The most restrictive liability of IL-2 therapy is its dose-limiting induction of vascular leak syndrome (VLS) that manifests as increased vascular permeability resulting in tissue edema and acute respiratory failure, hypotension leading to rhabdomyolysis, and ultimately hepatorenal syndrome and multiple organ failure (3, 4). A similar toxicity profile has been reported preclinically in nonhuman primate (NHP) models (5, 6). Multiple lines of evidence suggest that VLS stems primarily from the systemic release of IL-5 and induction of eosinophilia (3, 7, 8). It has been hypothesized that IL-5 is released from IL-2Rα chain expressing type 2 innate lymphoid (ILC-2) cells that reside in the endothelium of the vasculature (7). In addition, aldesleukin’s efficacy is dampened by its stimulatory effect in the activation and expansion of immunosuppressive regulatory CD4^+^ T cells (Treg), given its high affinity for engaging IL-2Rα, expressed at high levels on those cells (9). Finally, aldesleukin has a short systemic half-life (approximately 90 minutes; refs. 10, 11), limiting both its exposure and duration of pharmacodynamic (PD) effects.

Identification and development are ongoing for several next-generation IL-2 candidates for anticancer therapy that are better tolerated while exhibiting preferential action at immune Teff and NK cells with an optimized pharmacokinetic (PK)/PD relationship. Multiple design strategies have been pursued including fusion to modules intended to modify its exposure or direct homing, including bioconjugation [for recent reviews, see Overwijk and colleagues (12) and MacDonald and colleagues (13)]. We leveraged our Expanded Genetic Alphabet platform to design the IL-2 Synthorin molecule SAR-44425 (SAR’245 or pegenzileukin, previously THOR-707), with a targeted, irreversible polyethylene glycol (PEG) bioconjugate at position 65 (Pro65) of the polypeptide chain, through the addition of a new, genetically encoded amino acid (14). Site-specific pegylation confers several differential properties including fine-tuning of systemic clearance to increase exposure, enhancing accumulation and retention at the tumor site (Ma and colleagues; submitted for publication), reducing the risk of immunogenicity by masking potential immunogenic neoepitopes, and blockade of IL-2Rα chain engagement to prevent preferential activation of Tregs and cell types involved in induction of eosinophilia, improving its tolerability by decreasing the risk of VLS. SAR’245 showed single-agent antitumor activity in the B16F10 melanoma and the CT-26 colonic syngeneic mouse tumor models that was increased in combination with a programmed cell-death protein 1 (PD-1) inhibitory antibody in the CT-26 model (Ma and colleagues; submitted for publication; ref. 14).

Here, we describe the evaluation of the pharmacologic properties of this engineered IL-2 molecule leveraging synthetic biology, SAR’245 in NHP, and assess the pharmacologic profile of this precision-engineered IL-2 for optimal expansion of effector CD8^+^ T and NK cells without induction of VLS in a species relevant for assessment of the molecule’s potential therapeutic index in humans and cynomolgus monkeys. We demonstrate a significant extension of peripheral half-life in dosed animals, resulting in sustained exposure over time. The PK profile of SAR’245 supports a robust expansion of the target peripheral CD8^+^ T and NK cells in NHP, with minimal elevation of Treg cells. No eosinophilia or signs of VLS were detected at any of the dose levels tested, including up to 10-fold higher than that eliciting maximal expansion of peripheral target CD8^+^ T and NK cells, suggesting a therapeutic index not previously observed, or possible, with aldesleukin (5, 6, 8, 15, 16). In addition, *ex vivo* studies with human primary immune cells indicate the potential for safe combination of SAR’245 with checkpoint inhibitory antibodies in the clinic. *Ex vivo* assays using human peripheral blood mononuclear cells (PBMC) demonstrate that this engineered version of IL-2 phenocopies IL-2 pharmacologic effects on induction of CD8^+^ T- and NK-cell proliferation and cytolytic activity. More importantly and surprisingly, SAR’245 demonstrated reversal of CD8^+^ T-cell exhaustion *ex vivo* by promoting proliferation and enhancing cytolytic activity of those cells. Taken together, our findings support the ongoing clinical development of SAR’245 as an immuno-oncology drug candidate, which is currently underway for multiple cancer indications (NCT04009681; ref. 17).

## Materials and Methods

### Animal models

Purpose-bred cynomolgus monkeys (*Macaca fascicularis*) were sourced from licensed vendors and underwent standard quarantine periods prior to study initiation. Animal studies were conducted in AAALAC-accredited facilities at Charles River Laboratories under protocols approved by the Institutional Animal Care and Use Committee. Male cynomolgus monkeys aged 2 to 4 years and weighing up to 2 to 3 kg were used for the study.

### Human donors

Healthy donor leukocyte reduction systems (LRS) were purchased from Cell IDX or performed under contract by PrimityBio. PrimityBio purchased human blood samples through an Institutional Review Board (IRB) approval at Stanford University. The collection from healthy donors at Synthorx was approved by the IRB of The Scripps Research Institute (#177065). PBMCs were acquired from normal healthy donors who provided written informed consent and who were enrolled in a Sanofi Pasteur VaxDesign Co. (SP-VxD) apheresis study (protocol # 0906009; Pro00005129) and donor program reviewed and approved by Advarra Inc. and conducted in accordance with recognized ethical guidelines (including The Belmont Report, Nuremberg Code, Declaration of Helsinki). Blood collections were performed at OneBlood blood bank using standard techniques approved by their IRBs, and PBMCs were freshly isolated from apheresis at SP-VxD (18). Samples from HLA A*02:01 positive donors were purchased from AllCells, and enriched NK cells were purchased from AllCells and StemCell Technologies.

### Cell lines

K562 cells were purchased from the American Type Culture Collection. Cells were within 10 passages and cultured in complete RPMI (RPMI + 10% FBS + 1% penicillin/streptomycin).

### Test article and formulation

SAR’245 was manufactured at Synthorx. Lyophilized SAR’245 was first reconstituted with 0.1 mol/L acetic acid to the stock concentration, then diluted with 1× phosphate buffer saline (PBS; without Ca^2+^ and Mg^2+^) with 0.25% v/v cynomolgus monkey plasma. The dosing solution was individually prepared for each animal with its own plasma.

### pSTAT5 *ex vivo* assay

Blood cells from human and cynomolgus monkeys were freshly sourced. Fresh lymphocytes were treated with either hIL-2 or SAR’245 in a series dilution for 40 minutes. After the stimulation, samples were fixed and stained with a flow cytometry panel, which includes antibodies to identify CD8^+^ T, NK, and Treg cells, as well as pSTAT5 antibody (14).

### Surface plasmon resonance

Surface plasmon resonance (SPR) studies were performed under contract by Biosensor Tools LLC, as previously described (14). A detailed list of all compounds used in the SPR studies is provided in Supplementary Table S1.

### Animal study design

For the dose-range finding study, cynomolgus monkeys received once-weekly i.v. bolus injections of SAR’245 at doses of 0.03, 0.1, 0.3, and 1 mg/kg. The animals were necropsied 8 days after the last dose.

For the dose-scheduling study, animals received 0.1 mg/kg i.v. bolus injections of SAR’245 once a week (QW), every 2 weeks (Q2W), every 3 weeks (Q3W), or every 4 weeks (Q4W) for three dosing cycles. The vehicle group was dosed once a week for 3 weeks. Samples were collected for up to 1 month after the last dose and then animals were returned to the colony after study completion.

Clinical signs of toxicity were subjectively determined following standard procedures. Food consumption was qualitatively assessed on a daily basis and the body temperature was determined employing digital infrared thermometers at 24, 9, and 168 hours following each dose. The temperature was collected pretreatment and also 24 and 48 hours after each dosing.

### Plasma cytokine quantification with Luminex multiplex assay

Plasma samples collected from NHP studies were analyzed by Luminex multiplex assay, following the manufacturer’s guidelines. The detection limit for IFNγ, TNFα, IL-5, and IL-6 was 150 pg/mL. All cytokine analyses were conducted at Charles River Laboratories.

### Flow cytometry of NHP whole blood samples

Blood samples were collected to evaluate the effects of SAR’245 on immune cells with flow cytometry. Peripheral blood samples were treated for erythrocyte lysis and fixed with BD Phosflow Lyse/Fix buffer immediately after collection. Cells were blocked with human TruStain FcX (BioLegend) before staining for cell surface markers.

The following surface markers were used to identify immune cell types: T cells [CD3^+^ (BD Biosciences, clone SP34-2)], CD4^+^ helper T cells [CD3^+^/CD4^+^ (BioLegend, clone OKT4)], CD8^+^ T cells [CD3^+^/CD8^+^ (BD, clone SK1)], CD4^+^ Treg cells [CD3^+^/CD4^+^/CD25^+^ (BioLegend, clone M-A251)/FoxP3^+^ (BioLegend, clone 259D)], and NK cells [CD3^−^/CD7^+^ (BD, clone M-T701)]. Cells were permeabilized and stained internally with anti-pSTAT5 (Y694) antibody [BD, clone 47/Stat5(pY694)] and anti-Ki67 (Thermo Fisher, clone SolA15) antibodies. Samples were read using BD LSR Fortessa flow cytometer, and analysis was performed employing the FlowJo software v10.

### Measurement of plasma SAR’245 levels

Blood samples were collected in EDTA K3 tubes and processed per the manufacturer’s specifications for plasma separation. SAR’245 concentration in plasma was quantified with a sandwich ELISA method using a commercial antihuman IL-2 ELISA kit (Ab100566). Toxicokinetic parameters were extracted employing Pheonix WinNonlin v6.4, using a noncompartmental model.

### Allogeneic MLR assay

PBMCs from two normal healthy donors were used in each assay run. CD4^+^ T cells were isolated from one donor and monocytes were isolated from another donor using magnetic beads. Monocytes were transdifferentiated into monocyte-derived dendritic cells (monoDC) via addition of human IL-4 and GM-CSF to *ex vivo* cultures for 7 days. Mixed lymphocyte reaction (MLR) was performed by coculturing the isolated CD4^+^ T cells with the differentiated monoDCs at a ratio of 10:1 T cell to monoDC. CD4^+^ T cells and monoDCs were incubated for 5 days in the presence of serial dilutions of SAR’245 (48–30,000 ng/mL) with or without 5, 50, or 500 ng/mL of clinical grade pembrolizumab, nivolumab, or ultra-LEAF purified human isotype control IgG4 (BioLegend). Pembrolizumab alone and nivolumab alone treatments were tested in serial dilutions of 1 to 10,000 ng/mL. Culture supernatants were harvested on day 5 for ELISA analysis of IFNγ production (Abcam).

### Whole blood cytokine release assay

Fresh human whole blood from healthy donors was incubated with a broad titration range of SAR’245 as a single agent or in combination with a fixed concentration of pembrolizumab (90 μg/mL), nivolumab (127 μg/mL), or Ultra-LEAF purified human IgG4 isotype control (BioLegend, 500 ng/mL) for 24 hours. An anti-CD3 antibody (clone UCHT1, BioLegend) was used as a positive control. Cytokines released from whole blood samples into the cultures were measured using the MSD U-plex kit (Meso Scale Diagnostics) for six analytes (IFNγ, IL-4, IL-5, IL-6, IL-8, and TNFα). The range of concentrations of SAR’245 (0.2–4.5 μg/mL) was selected based on the NHP exposure data. rhIL-2 (R&D Systems; # 202-IL-500) was also included in this assay. The titration range of rhIL-2 (0.03–0.8 μg/mL) covers the projected maximum drug concentration (*C*_max_) of aldesleukin clinical dose (19). The concentration of pembrolizumab at 90 μg/mL was selected based on the *C*_max_ level reported in patients receiving a 200-mg dose of the antibody (20). Likewise, the concentration of nivolumab at 127 μg/mL was based on the published plasma *C*_max_ in patients receiving a 360-mg dose (21).

### Flow cytometry of *in vitro* cultured PBMC

PBMCs were enriched from whole blood using SepMate50 tubes (Stemcell Technologies), following the manufacturer’s guidelines. PBMCs were washed and cultured in complete RPMI [RPMI + 10% FBS + 1% penicillin/streptomycin] with rhIL-2 (Gibco #PHC0023 or #PHC0027) or SAR’245 and cultured for 5 days with or without 1 mg/mL CMV HLA A*02:01 peptide (MBL International). After 5 days, the PBMCs were stained with Zombie Red (BioLegend), following the manufacturer’s guidelines. Cells were then stained with a cocktail of antibodies including TruStain FcX (BioLegend), HLA A*02:01 CMV pp65 iTAg tetramer in PE (MBL International), and antigen-presenting cell (APC) antihuman CD25 (M-A251) for 30 minutes at room temperature. PBMCs were washed with PBS + 0.5% BSA, treated with fixation buffer (BioLegend) for 15 minutes, washed with 1× permeabilization buffer (BioLegend), and stained with another cocktail of antibodies for 30 minutes. The panel included APC/Cy7 antihuman CD3 (UCHT1), PE/Cy7 antihuman CD4 (RPA-T4), PerCP/Cy5.5 antihuman CD8 (RPA-T8), eFluor 506 anti-Ki67 (SolA15), FITC antihuman Perforin (B-D48), and BV421 antihuman Granzyme B (QA18A28). CMV^+^ CD8^+^ T cells are defined as CD3^+^CD8 tetramer^+^, and NK cells are defined as CD3^−^CD56^+^ perforin-positive cells.

### Killing assay

PBMCs or enriched NK cells were labeled with Cell Trace Yellow, following the manufacturer’s guidelines, and primed overnight with rhIL-2 (Gibco #PHC0023 or #PHC0027) or SAR’245. A small aliquot of PBMC was stained with BV421 antihuman CD56 (HCD56) for 15 minutes to calculate the frequency of NK cells. Primed cells were cocultured with Cell Trace Violet labeled K562 cells at a ratio of 3:1, 1:1, 1:3, and 1:9 for 4 hours at 37°C. Cells were stained with 7-AAD or Live/Dead NIR and acquired on an Attune NxT flow cytometer (ThermoFisher). The frequency of dead K562 cells is reported.

### Phospho flow cytometry

Phospho flow cytometry studies were performed at PrimityBio. Human LRS cones were received the same day as drawn from healthy donors. The LRS chambers were drained into a 50-mL conical tube and the volume was adjusted to 20 mL using PBS. A measured quantity of 90 μL of the stock was then aliquoted into the relevant wells of a 96-well plate. The plate was warmed to 37°C for 15 minutes prior to the addition of non-Treg CD4 EC90 rhIL-2 or SAR’245 for 45 minutes. Potency in non-Treg CD4 cells was selected because these cells show the lowest response to SAR’245, to then maximize response in NK and CD8^+^ T cells. At the end of the incubation period, the erythrocytes were lysed, and the cells were fixed in BD Lyse/Fix Buffer (Beckton Dickinson, Cat. # 558049) for 10 minutes, then centrifuged at 450 *g* for 5 minutes to pelletize. The pelleted cells were washed with PBS + 0.5% BSA and stored at −80°C until analysis. The cells were stained with a cocktail of antibodies including CD3, CD4, CD8, CD16/CD56, CD19, CD25, and CD127 and barcoded to include >100 phospho antibodies and total proteins (as shown in Supplementary Table S2).

The median Fluorescence Intensity (MFI) of each target population was exported. The fold change in MFI relative to untreated control was calculated for NK cells (CD3^−^CD56^+^), CD8^+^ T cells (CD3^+^CD8^+^), and CD4^+^ Treg cells (CD3^+^CD4^+^CD127^−^CD25^+^). A fold change ≥1.1 is shown.

### CD8^+^ T-cell exhaustion assay

Autologous donor CD8^+^ T cells were isolated from frozen PBMCs by negative magnetic bead selection according to the manufacturer’s instructions (STEMCELL Technologies; EasySep) and APCs were generated as previously described (18). The isolated CD8^+^ T cells were cocultured with autologous dendritic cells previously primed with antiviral HLA-restricted peptides (BioSynthesis, Inc.) at a ratio of 60:1 and incubated at 37°C and 5% CO_2_ in *X-VIVO* 15 serum-free media (Lonza) for 12 to 14 days. The exhausted CD8^+^ T cells were developed by repeated Ag exposure to the appropriate peptide over the 12 to 14-day coculture. Phenotypic and functional markers of T-cell exhaustion were evaluated on Ag-specific CD8^+^ T cells marked by HLA Class I-restricted pentamers (Proimmune Ltd.) via flow cytometry (22).

Exhausted CD8^+^ T cells from 12 to 14-day cocultures were labeled with carboxyfluorescein succinimidyl ester (CFSE) using protocols established at SP-VxD and restimulated with fresh autologous APCs pre-primed with the appropriate HLA Class I-restricted peptide. Culture wells were treated with various doses of Synthorin molecule, recombinant human cytokine, or peptide alone at 37°C in 5% CO_2_ for 7 days. Functional CD8^+^ T cells from 12 to 14-day cocultures were also stimulated with peptide-pulsed DC as a control. Proliferation was assessed by flow cytometry evaluating the number of HLA-pentamer^+^ Ag-specific CD8^+^ T cells and the extent of CFSE dye dilution. Phenotypic markers of T-cell exhaustion were evaluated on Ag-specific CD8^+^ T cells marked by HLA Class I-restricted pentamers.

Supernatants from the various 7-day restim CD8^+^ T-cell culture conditions were analyzed for IFNγ and TNFα using the Milliplex Human Cytokine Custom 10 Plex detection system (EMD Millipore), per the manufacturer’s protocol, and cytokine responses were measured on BioPlex/Luminex Systems and the data acquisitioned in Bio-Plex Manager Software (Bio-Rad; ref. 22).

### Statistics

Statistical analysis was performed using GraphPad Prism. Studies utilized one-way or two-way ANOVA, where indicated, with Dunnett *post hoc* test for statistical analysis. Treatment groups are compared with vehicle control. Significance is defined as *P* < 0.05.

### Data availability

The datasets generated and/or analyzed during these studies are available from the corresponding author upon reasonable request.

## Results

### SAR’245 exhibits differential pharmacology on Teff and NK cells relative to Tregs in both humans and NHP

We designed SAR’245 to have reduced affinity for the IL-2Rα chain, while retaining engagement of IL-2Rβ (14). SPR analysis shows that the equilibrium binding constants of rhIL-2 to the cynomolgus IL-2Rα and β chains are essentially identical to those observed for the human receptor chains (Supplementary Table S3; Supplementary Fig. S1A and S1B). In contrast, SAR’245 retains IL-2Rβ binding activity while not engaging the IL-2Rα chain of human or NHP (Supplementary Table S3; Supplementary Fig. S1A and S1B). The affinity of SAR’245 for human and NHP IL-2Rβ is roughly four to five folds lower than native rhIL-2 (Supplementary Table S3; Supplementary Fig. S1B). In this regard, SAR’245 displays a moderately reduced on-rate at IL-2Rβ, probably because the PEG moiety affects the diffusion coefficient.

We employed flow cytometry to study the activation of human and NHP IL-2 receptors at target and nontarget cells *ex vivo* by monitoring pSTAT5 formation. As expected, CD4 Treg cells have significantly higher IL-2Rα (CD25) intensity on the surface of CD4 Treg cells, compared with CD8^+^ and NK cells, in both human and monkey PBMC cells (Supplementary Fig. S1C and S1D). In addition, rhIL-2 demonstrated similar potency for induction of pSTAT5 signaling in primary cells of cynomolgus monkeys relative to human PBMCs (Supplementary Table S4). In contrast, although SAR’245 was similarly active on human and monkey immune cells, its potency was overall reduced compared with rhIL-2. Importantly, SAR’245 stimulated STAT5 phosphorylation in Tregs, NK, and CD8^+^ Teff cells with comparable potency (Supplementary Table S4).

The observed similarities in the potency of unconjugated IL-2 and our pegylated version of IL-2 on primary human and NHP CD8^+^ T and NK cells suggest that cynomolgus monkeys are appropriate for preclinical evaluation of the pharmacologic effects, safety, and tolerability of SAR’245. Moreover, this conclusion aligns with previous studies of aldesleukin demonstrating that findings in NHP were translatable to humans (5, 6, 15).

### NHPs dosed with SAR’245 did not develop VLS

SAR’245 administered via i.v. bolus to cynomolgus monkeys did not cause mortality at doses up to 1 mg/kg, which is 10 folds greater than the dose eliciting maximal expansion of Teff and NK cells and nearly 2.5× above the lethal dose of aldesleukin in NHP (15). No SAR’245-related changes in food consumption, body temperature, or organ weights were noted (Supplementary Fig. S2A–S2C) up to 1 mg/kg, except for an elevation in spleen weight at 1 mg/kg, which is consistent with increased lymphocytic traffic to that organ. These results are different from aldesleukin, which reportedly induces increase in spleen, lung, and kidney weights (15). Supplementary Fig. S2D shows peripheral IL-5 levels throughout the study (placebo vs. SAR’245 1 mg/kg).

**Figure 1:**
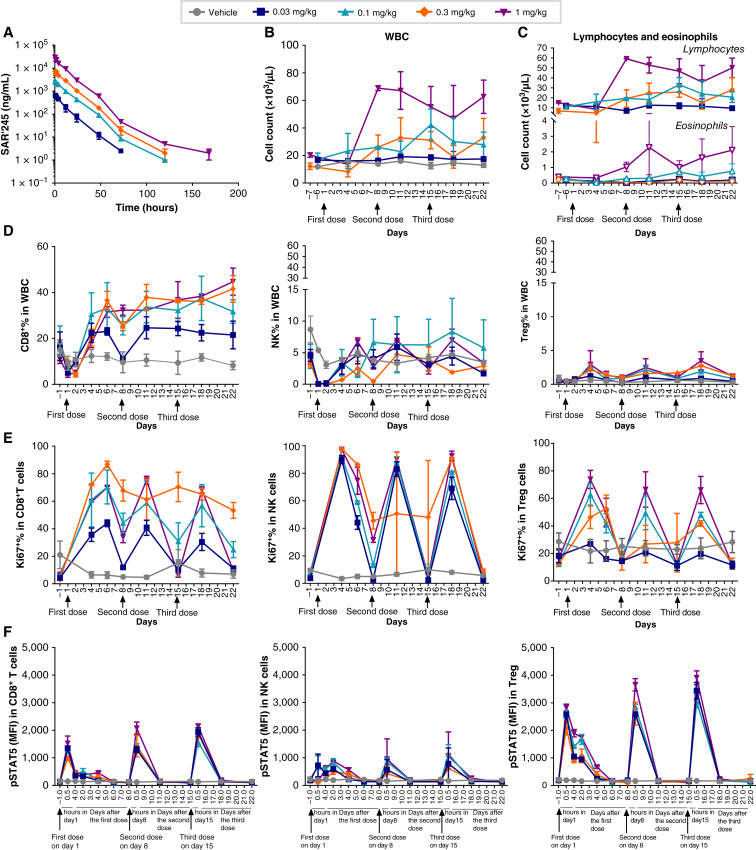
Pharmacokinetic profile of SAR’245 in a dose-range finding study in cynomolgus monkeys. SAR’245 was given i.v. at 0.03, 0.1, 0.3, and 1 mg/kg vs. vehicle once a week for three dosing cycles (days 1, 8, and 15). **A,** SAR’245 concentration was measured from plasma samples collected from dosed treated animals following the first dose. Mean values are shown from two animals in each group. **B,** Changes in WBC in response to repeat dosing with SAR’245. **C,** Cell count changes in response to SAR’245 dosing for lymphocytes (•) and eosinophils (◦). **D,** Percentage of CD8 T, NK, and CD4 Treg cells in peripheral blood. **E,** Percentage of Ki67 expression in CD8 T, NK, and CD4 Treg cells in the peripheral blood. **F,** MFI of pSTAT5 in CD8 T, NK, and CD4 Treg cells in peripheral blood. Mean ± SEM. *N* = 2. MFI, median fluorescence intensity; NK, natural killer; pSTAT5, phosphorylated signal transducer and activator of transcription 5.

Maximum concentrations of SAR’245 were generally observed at the first sampling (0.5 hours post-dose; Fig. 1A; Supplementary Fig. S3A). The mean half-life (*t*_½_) and clearance (CL) values ranged from 9.35 to 11.2 hours and 2.59 to 4.19 mL/hours/kg, respectively (Supplementary Table S5). Mean volume of distribution (*V*_d_) values ranged from 35.7 to 61.3 mL/kg, suggesting that SAR’245 is restricted primarily to the vasculature (Supplementary Table S5), consistent with previous observations for biologic drugs (23). Systemic exposure to SAR’245, as measured by *C*_max_ and area under the concentration–time curve of 0 to 168 hours (AUC_0–168 hours_), increased proportionally with dose on day 1.

Aldesleukin is known to induce elevations in lymphocytes and eosinophils in both species at comparable dosing levels, and it is hypothesized that VLS is mediated by eosinophil expansion (24, 25). As shown in Fig. 1B (and Supplementary Fig. S3B), following i.v. bolus administration of three repeat doses of SAR’245, white blood cell (WBC) counts increased in peripheral blood samples in a dose-proportional fashion. Although the lowest tested dose of SAR’245 (0.03 mg/kg) showed minimal expansion of WBCs in the NHP model, doses of 0.1 mg/kg and higher showed a transient drop in peripheral blood lymphocyte counts at 72 hours post-dose (day 4), suggesting IL-2-mediated extravasation, followed by subsequent cell expansion (26). SAR’245-induced lymphocyte counts increased after each dose until the end of the study (day 22; Fig. 1C). The degree of expansion was similar at the 0.3 and 1 mg/kg dose levels.

No significant elevation in eosinophil counts was observed at dose levels up to 0.3 mg/kg; a moderate increase was detected at the highest dose of 1 mg/kg in one of two animals (Fig. 1C; Supplementary Fig. S3C), which seems lower relative to eosinophil expansion levels reported in patients and monkeys treated with high-dose rhIL-2 (16, 24). In addition, cytokines that are associated with cytokine release syndrome (CRS)—IL-6, TNFα, and IFNγ (27, 28)—were all below the limit of detection. We also monitored peripheral IL-5 because of its elevation in patients developing VLS in response to aldesleukin administration (8). It was induced in moderate levels in the same one animal dosed at 1 mg/kg SAR’245 (Supplementary Fig. S2D) that showed an increase in eosinophils (Fig. 1C). Similar to humans, eosinophilia was observed in NHPs dosed with aldesleukin, with manifestations of VLS that were dose-proportional, reaching lethality at 0.428 mg/kg (5, 6, 15).

### SAR’245 preferentially expands CD8^+^ T and NK cells in NHP

The effects of SAR’245 on the activation and expansion of peripheral CD8^+^ T, NK, CD4^+^ T, and Treg cells were analyzed by flow cytometry. As observed with peripheral blood lymphocytes (Fig 1C), CD8^+^ T-cell levels transiently declined, presumably due to exit from the circulation, and subsequently expanded at all dose levels (Fig. 1D; Supplementary Fig. S3D). Of note, the effects of IL-2 on *in vivo* cell trafficking are well documented and probably key to orchestrating antitumoral responses (29). The increase in CD8^+^ T-cell counts in peripheral blood peaked at 5 days post-dose, then dropped by day 8 prior to the second dose (Supplementary Fig. S4A). Following the second and third dose of SAR’245, peripheral blood CD8^+^ T cells expanded and remained elevated up to the end of the study (Supplementary Fig. S4A). NK cells followed a similar post-dosing pattern with transient reduction followed by the expansion that peaked after approximately 5 days, returning to baseline after each dose (Fig. 1D; Supplementary Fig. S4A). Given the overall low percentage of peripheral CD4^+^ Treg cells in this study, the observed level of CD4^+^ Treg increase was minimal compared with the expansion of CD8^+^ T and NK cells (Fig. 1D; Supplementary Fig. S4A). Compared with CD8^+^ T cells, no noticeable expansion of total CD4^+^ T cells was observed at or below 0.3 mg/kg, with a small increase in total CD4^+^ at 1 mg/kg (Supplementary Fig. S4B and S4C).

Ki67, a nuclear factor required for chromosomal segregation as cells enter mitosis (30), serves as a molecular PD marker for cell proliferation. Ki67 positivity was observed in all three cell populations monitored (CD8^+^ T, NK, and Treg) following each dose of SAR’245 at all dose levels (Fig. 1E; Supplementary Fig. S3E). The percentage of Ki67^+^ T cells and NK peaked over 3 to 5 days following each dose, and subsequently dropped to different extents depending on the dose level, mirroring the pattern observed in peripheral blood in these groups (Fig. 1D). As expected, Ki67 peaks preceded increases in cell numbers.

Peripheral blood pSTAT5 levels were also evaluated as a more proximal marker of IL-2R occupancy and early signaling. SAR’245 induced a rapid and sharp rise of pSTAT5 in peripheral blood CD8^+^ T, NK, and Treg cells at 0.5 hours following each dose (Fig. 1F; Supplementary Fig. S3F), with pSTAT5 levels dropping back to baseline within 3 days in all three cell populations.

Taken together, the time course of induction of PD markers as analyzed above revealed a sequence induced by SAR’245 that aligns with expectations for native IL-2. pSTAT5 was promptly induced after each dosing event, which subsequently translated into cell proliferation as indicated by increases in Ki67 positivity and ultimately by increases in CD8^+^ T and NK cells as well as Tregs. However, Treg numbers increased very modestly and transiently, around 3 to 5 days following each dose. Overall, elevations in CD8^+^ T cells seemed more sustained compared with the expansion in NK cells, which were more robust than Treg cells.

### Pharmacodynamic responses in NHP are comparable with different SAR’245 dosing schemes

A backbone therapy for immuno-oncology should ideally offer flexibility with regard to the dosing scheme, given the multiple combination agents and dosing schedules employed in the clinic. SAR’245 was administered by i.v. bolus at 0.1 mg/kg on a QW, Q2W, Q3W, or Q4W dosing schedule for a total of three doses. No signs of safety concerns were observed (Supplementary Fig. S5A–S5C), including no significant changes in body weight or body temperature (Supplementary Fig. S5C).

**Figure 2:**
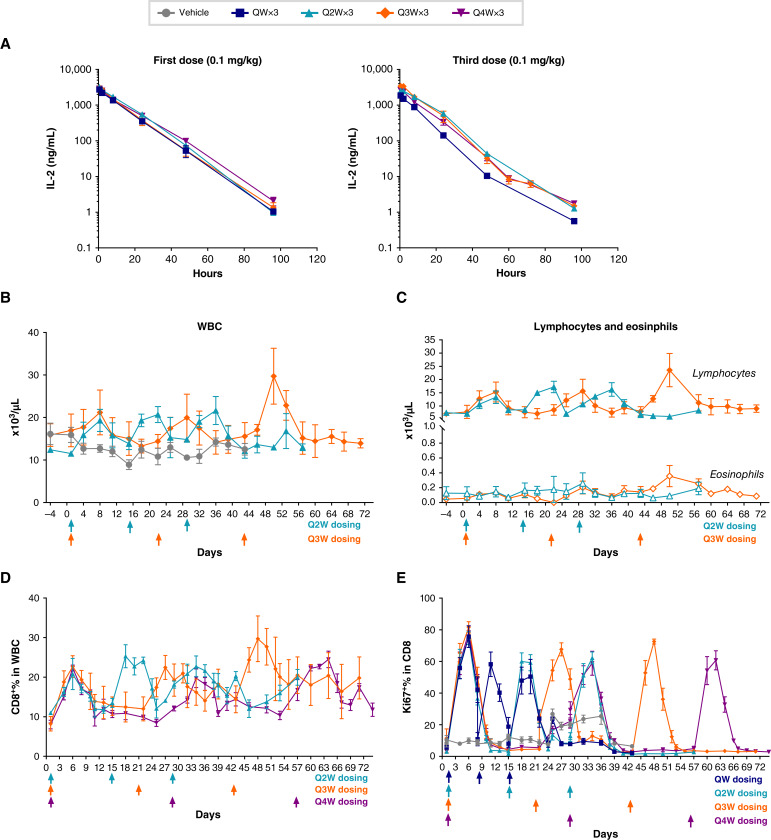
Pharmacokinetic and pharmacodynamic profiles of SAR’245 in NHP following variable dosing schedules. SAR’245 administered at 0.1 mg/kg i.v. to cynomolgus monkeys on a QW, Q2W, Q3W, or Q4W dosing schedule for three repeat dosing rounds. **A,** Plasma PK analysis was performed after the first and the third i.v. bolus doses of SAR’245. **B,** Changes in WBC from Q2W and Q3W groups in response to repeat dosing with SAR’245. **C,** Changes in lymphocyte and eosinophil counts from Q2W and Q3W groups. **D,** Changes (%) in CD8^+^ T cells in peripheral blood following each repeated dose in Q2W and Q3W dose groups. **E,** Ki67^+^ % in CD8 T in peripheral blood following each of three repeat doses of SAR’245 in all dose groups. Reported as mean ± standard error of the mean (SEM) of *N* = 3 animals.

Supplementary Table S6 shows the PK parameters of SAR’245 and Fig. 2A depicts the plasma concentration (mean ± SEM) following the first and the third i.v. bolus doses. As noted before, the maximum plasma concentration of SAR’245 was observed at 0.5 hour post-dose (the first measured time point after dosing) in all animals at all dosing schedules. After the third dose, time to peak drug concentration (*T*_max_) was observed at 0.5 to 2 hours post-dose in all animals analyzed. The *C*_max_ and total drug exposure (AUC_0–168 hours_) following the first and third doses were comparable for the Q2W and Q3W dosing schedules (Supplementary Table S6). Following three repeat Q2W, Q3W, and Q4W doses, the CL, *V*ss, and *t*_½_ values were consistent between the first and third doses. In the QW group, *t*_½_ and *V*ss were also consistent between the first and the third doses (Supplementary Table S6).

As shown in Fig. 2B, SAR’245 at 0.1 mg/kg on QW, Q2W, Q3W, or Q4W dosing schedules increased WBC counts in peripheral blood samples. WBC expansion was similar across all dosing schedules with lymphocytes representing the majority of the change in WBC count (Fig. 2B and C; Supplementary Fig. S3B and S3C). As observed before with QW dose frequency, WBC and lymphocyte counts progressively increased and remained elevated with each dose before returning to pre-dose levels around 10 days after the third dose (Supplementary Fig. S5A and S5B). The other dosing schedules (Q2W, Q3W, and Q4W) showed both WBCs and lymphocytes peaking 7 days after each dose and returning to control or pre-dose levels prior to the subsequent dose (Fig. 2B and C; Supplementary Fig. S5A and S5B). The degree of lymphocyte expansion was similar across all dosing schedules (Fig. 2B; Supplementary Fig. S2B). No significant elevation in eosinophil counts was observed with any of the dosing regimens (Fig. 2C; Supplementary Fig. S5B). We did not observe changes in body temperature, body weight, or in hemodynamic parameters with any dosing schedule including the most frequent one of QW dosing (Supplementary Fig. S5C).

The QW dose group showed a similar trend of CD8^+^ T expansion as observed in the dose range-finding study, with the percentage of peripheral CD8^+^ T cells peaking 5 days following the first dose, showing a slight drop by day 8 before the second dose, and subsequently remaining elevated up to day 22 (Fig.1D). Q2W, Q3W, and Q4W dose groups showed a similar PD trend for CD8^+^ T-cell expansion in response to repeat dosing (Fig. 2D). For these three groups, CD8^+^ T cells reached maximal expansion 5 days after each dose, taking 7 to 10 days to return to the baseline (Fig. 2D). In all dosing schedules, peak levels of CD8^+^ T cells after each repeat dosing were similar to the first dose. A similar pattern was observed for Ki67 with the proportion of Ki67-positive CD8^+^ T cells peaking 3 to 5 days following each dose (Fig. 2E). This indicates sustained drug activity with repeat-dosing for all regimens. Similarity in PD response among the Q2W, Q3W, and Q4W dosing schedules relative to QW suggests that the maximum PD effect is mainly driven by the dose (“pulse” pharmacology that does not require constant receptor occupancy). Together these results suggest that SAR’245 is amenable to flexible dose regimens in the setting of combination therapy with other immune-oncology agents.

### Additive activity of SAR’245 in combination with anti-PD-1 antibodies in T-cell receptor–stimulated human primary cells

**Figure 3:**
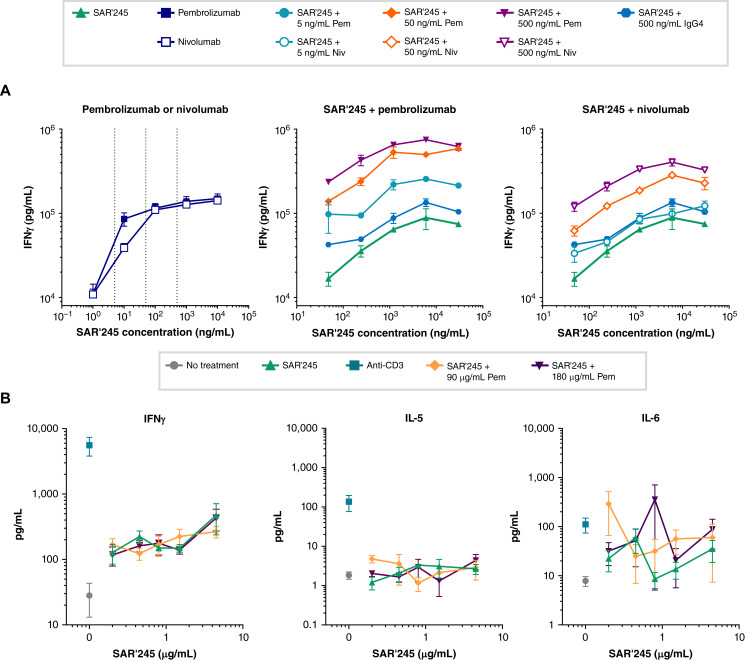
SAR’245 alone and in combination with anti-PD-1 antibodies enhances IFNγ secretion from activated T cells. **A,** Purified CD4 T cells were cocultured with allogeneic monoDCs in the presence of varying concentrations of SAR’245, nivolumab, or pembrolizumab. Supernatants were collected after 5 days of culture and measured for IFNγ production by ELISA. Representative data from multiple healthy donor pairs are shown (*N* = 3). **B,** Whole blood samples were coincubated with varying concentrations of SAR’245 alone or in combination with 90 or 180 μg/mL pembrolizumab for 24 hours. Cytokine released into culture supernatants was measured by MSD (IFNγ, TNFα, IL-5, IL-6, IL-4, and IL-8). TNF α, IL-4, and IL-8 are not shown. Plotted as average ± SEM. *N* = 4 donors. IFNγ, interferon gamma.

The ability of SAR’245 to promote T-cell activation was evaluated *ex vivo* using freshly isolated T-cell receptor (TCR)–activated human T cells, employing an allogeneic MLR model. As shown in Fig. 3A, SAR’245 applied as a single agent in MLR cocultures induced concentration-dependent release of IFNγ production.

Checkpoint inhibitory anti-PD-1 monoclonal antibodies antagonize the inhibitory interaction between PD-1 and its ligand PD-L1 on antigen-presenting tumor cells. Therefore, we explored the potential use of SAR’245 in combination with therapeutic anti-PD-1 antibodies in the MLR assay system. In agreement with previous reports (31), Fig. 3A shows that both nivolumab and pembrolizumab induced a dose-dependent enhancement of IFNγ release as single agents, but the addition of SAR’245 enhanced IFNγ secretion, compared with each single agent. This additive effect was not seen when human IgG4 was used as the control antibody. Data from this *ex vivo* assay were consistent with the observation in the CT26 syngeneic mouse colonic tumor model, in which an enhanced antitumor activity was observed in the combination treatment group (SAR’245 plus anti-PD-1 antibody) compared with the single-agent treatment (Ma and colleagues; submitted for publication). These results suggest the potential for combined use of SAR’245 with anti-PD-1 antibodies. Indeed, SAR’245 is currently being tested in the clinic in combination with pembrolizumab or cemiplimab in phase 1b/2a clinical studies (Clinicaltrials.gov: NCT04009681, NCT05104567, NCT05179603, NCT04913220, NCT05061420, NCT04914897).

### SAR’245 alone or in combination with anti-PD-1 antibodies does not elicit release of cytokines associated with CRS from human whole blood

Aldesleukin dosed as a single agent in patients with cancer has been associated with manifestations of CRS (3), raising the concern that combining IL-2 with checkpoint inhibitors may lead to increased severity of CRS. We preliminarily addressed this potential liability by employing a preclinical whole blood cytokine release model to assess the effect of SAR’245 as a single agent and in combination with an anti-PD-1 antibody on proinflammatory cytokine secretion.

Fresh blood samples from healthy donors were incubated with a range of SAR’245 or rhIL-2 concentrations for 24 hours, with or without nivolumab or pembrolizumab. The positive control was an antihuman CD3 antibody, and the negative control was an hIGg4 isotype antibody. We monitored six cytokines secreted into the culture media (IFNγ, IL-5, IL-6, TNFα, IL-4, and IL-8). Except for IL-5, which reports on innate lymphoid cell and eosinophil activation triggering VLS (7), these cytokines are among those consistently elevated in the serum of patients with CRS (27, 28). Incubation of human whole blood cells with anti-CD3 resulted in robust cytokine production, whereas incubation with SAR’245 induced only a moderate increase of IFNγ, which was concentration-dependent (Fig. 3B). Increase of IFNγ with SAR’245 is comparable to that observed with rhIL-2 (Supplementary Fig. S6). This response is expected from the pharmacologic effect of IL-2, which induces IFNγ production by CD8^+^ T and NK cells. The addition of pembrolizumab or nivolumab did not result in a noticeable change in the profile of IFNγ (Supplementary Fig. S6). SAR’245 as a single agent and in combination with pembrolizumab did not induce significant release of IL-5, IL-6, or TNFα (Fig. 3B; Supplementary Fig. S6). These results show the potential for an improved drug safety profile of SAR’245 when used as a single agent and in combination with SAR’245 and anti-PD-1 drugs.

### SAR’245 promotes proliferation and cytotoxicity of memory CD8^+^ T and NK cells

**Figure 4:**
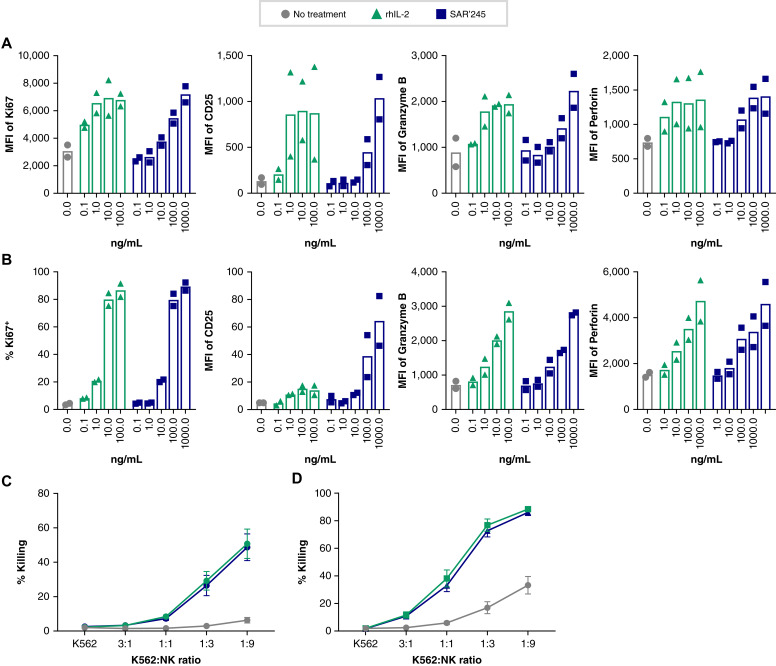
SAR’245 promotes CD8 T- and NK-cell proliferation and cytotoxicity *in vitro*. **A,** CMV+ donors were cultured with CMV peptide and rhIL-2 or SAR’245 for 5 days. The MFI of Ki67, CD25, perforin, and granzyme B on CMV+ CD8 T cells is reported. *N* = 2 donors. Data representative of two or more independent experiments. **B,** NK cell expression of Ki67, CD25, perforin, and granzyme B after a 5-day culture with rhIL-2 or SAR’245. *N* = 2 donors. Data representative of two or more independent experiments. **C,** Bulk PBMC or **D**, Enriched NK cells were primed overnight with rhIL-2 or SAR’245. Cells were then incubated with K562 target cells for 4 hours and percentage killing was measured. *N* = 3 donors. Data representative of one or more independent experiments. Data are shown as average ± SEM. MFI, median fluorescence intensity; NK, natural killer; rhIL-2, recombinant human interleukin-2.

Following on our work demonstrating that SAR’245 increased Teff cell activity (Ma and colleagues; submitted for publication), we sought to address whether our precisely pegylated IL-2 molecule could phenocopy IL-2 pharmacology for sustained proliferation of Teff and NK cells and for enhanced cytolytic capacity. Accordingly, we followed Ki67 and markers of activation in antigen-engaged CD8^+^ T cells and in NK cells from healthy donors treated with SAR’245. Figure 4A and B shows that like IL-2, SAR’245 promotes expression of Ki67 and CD25 (a marker of exposure to IL-2 and TCR activation) and of the cytolytic products granzyme B and perforin. Figure 4C and D shows that these biomarker changes translated into an increase in NK cell cytolytic capacity that closely phenocopies native rhIL-2.

**Figure 5:**
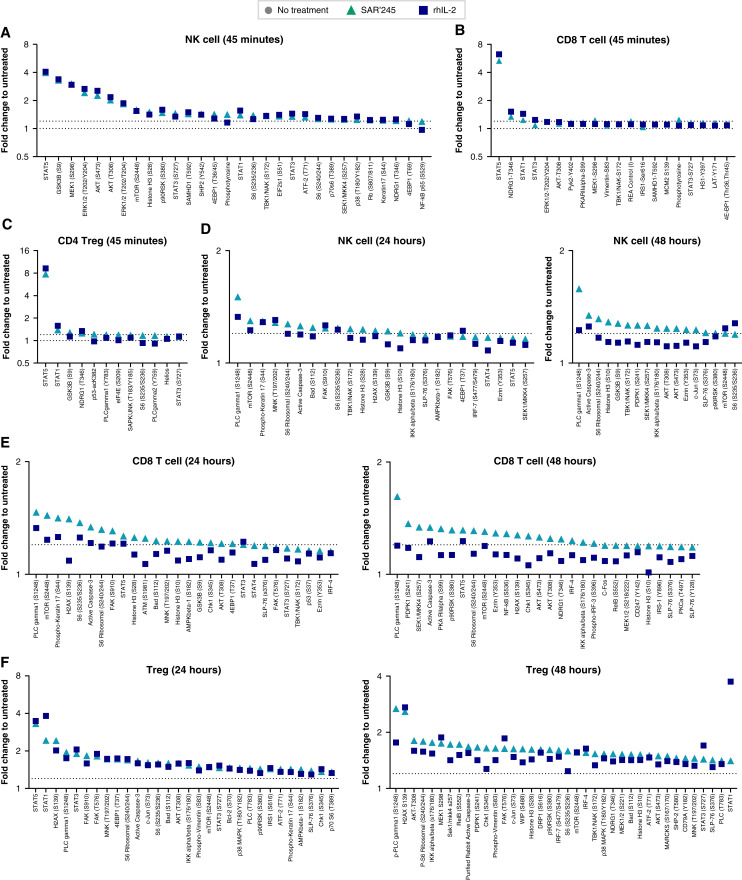
SAR’245 drives signaling pathways similar to rhIL-2. Bulk PBMC were treated with high-dose rhIL-2 or SAR’245 for 45 minutes and stained with a panel of 120 phosphoproteins. The fold change in MFI from untreated wells at (**A–C**) 45 minutes, (**D–F**) 24 or 48 hours was calculated and sorted from high to low in (**A** and **D**) NK cells, (**B** and **E**) CD8 T cells, and (**C** and **F**) CD4 Tregs. Dashed lines at 1 and 1.2. *N* = 1 donor. Data representative of one or more independent experiments. MFI, median fluorescence intensity; NK, natural killer; rhIL-2, recombinant interleukin-2; Treg, regulatory T cell.

The pharmacologic similarity of SAR’245 to IL-2 was further confirmed by fingerprinting its effect on the phosphoproteome of CD8^+^ T, NK, and Treg cells. In NK cells, SAR’245 followed, with remarkable precision, the phosphoproteomic changes observed with IL-2 stimulation (Fig. 5A). In CD8^+^ T and Treg cells, the major induced phosphoprotein, as expected, was pSTAT5, and quantitative values were almost identical for SAR’245 relative to rhIL-2 (Fig. 5B and C). Interestingly, and as expected from the lower consumption rate of SAR’245 versus native IL-2 in these cell cultures (Ma and colleagues; submitted for publication), phosphoproteins induced by SAR’245 were more persistent at 24 and 48 hours post-induction for NK and CD8^+^ T cells (Fig. 5D and E). For Treg cells, most phosphoproteome markers remain relatively low except pSTAT5, with a sustained effect observed with SAR’245 at 48 hours relative to IL-2 (Fig. 5F). Taken together, these data suggest that our modified not-alpha IL-2 phenocopies the effect of the unmodified lymphokine on CD8^+^ T and NK cell populations, the intended target populations for achieving antitumoral effects.

**Figure 6:**
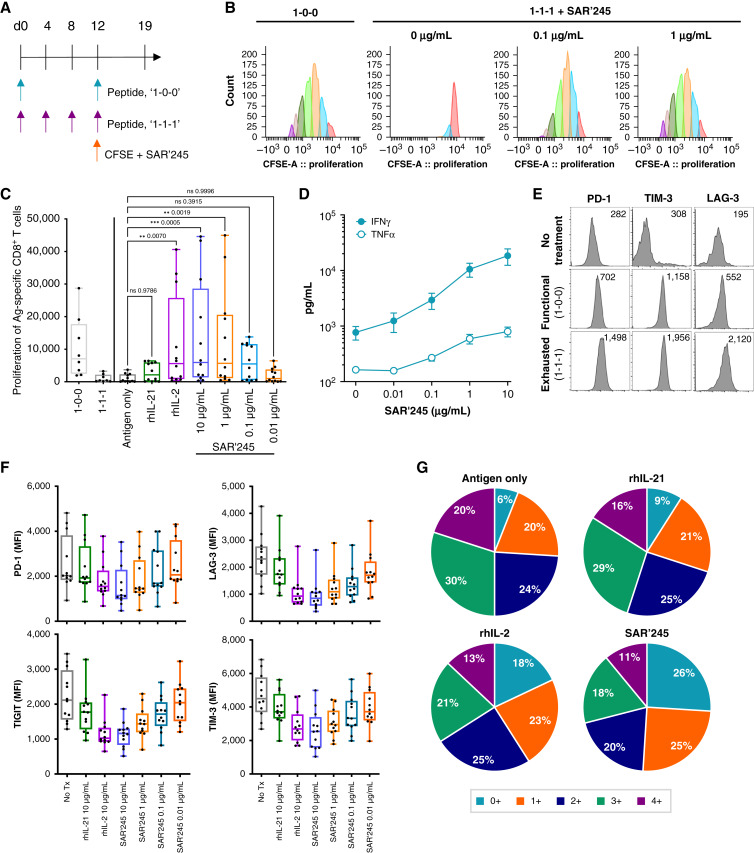
SAR’245 promotes activation of exhausted CD8 T cells. **A,** CD8 T cells were cocultured with peptide (pool) pulsed dendritic cells for 12 days. Freshly pulsed dendritic cells (DC) were added every 4 days to drive exhaustion. Cells were then labeled with CFSE and incubated with fresh peptide-pulsed DC in the presence of 10 μg/mL rhIL-21, 10 μg/mL rhIL-2, a titration of SAR’245 or peptide-pulsed DC alone (no treatment) for an additional 7 days. **B,** Representative CFSE plots; **C,** average proliferation; and **D,** cytokine secretion on day 19 of culture are shown. **E,** Representative histogram plots of PD-1, TIM-3, and LAG-3 expression by tetramer-specific CD8 T cells 12 days after no stimulation or repeated peptide stimulation. Number indicates MFI. **F,** MFI of PD-1, LAG-3, TIM-3, and TIGIT of exhausted CD8 T cells after 7 days treatment with SAR’245. *N* = 12 donors. Data are shown as average ± SEM. **G,** Percentage of exhausted CD8^+^ T cells expressing 0–4 markers of exhaustion (PD-1, LAG-3, TIM-3, and TIGIT) after 7 days treatment with rhIL-21, rhIL-2, or SAR’245 at 10 mg/mL. *N* = 12 donors. Significance was calculated by two-way ANOVA. CFSE, carboxyfluorescein succinimidyl ester; DC, dendritic cell; Ig, immunoglobulin; ITIM, immunoreceptor tyrosine-based inhibition motif; LAG-3, lymphocyte-activation gene 3; MFI, median fluorescence intensity; PD-1, programmed cell-death protein 1; rhIL, recombinant human interleukin; TIGIT, T-cell immunoreceptor with Ig and ITIM domains; TIM-3, T-cell immunoglobulin and mucin domain 3.

In the tumor microenvironment, CD8^+^ T cells have been shown to be functionally exhausted with impaired proliferation, secretion of inflammatory cytokines, and expression of markers such as PD-1, Tim3, Lag3, Slc16a11, CD49b, Tox, and others (32–34). *In vitro*, functionally exhausted CD8^+^ T cells can be induced through repeat stimulation with presented peptides (Fig. 6A), which is an experimental system we employed for exploring the effects of SAR’245. Repeat peptide stimulation produced CD8^+^ T cells that are deficient in their ability to proliferate or produce cytokine and that express high levels of PD-1, Tim3, and Lag3 (Fig. 6B–F). When exhausted CD8^+^ T cells were treated with SAR’245, their proliferative capacity and secretion of IFNγ and TNFα were restored (Fig. 6B–D). In contrast to rhIL-21, which did little to promote proliferation of exhausted CD8^+^ T cells at a similar dose level, SAR’245 also reduced the expression levels of PD-1, LAG-3, TIM-3, and TIGIT on exhausted CD8^+^ T cells (Fig. 6F). Additionally, SAR’245 decreased the percentage of CD8^+^ T cells coexpressing three or four inhibitory receptors while increasing the percentage of CD8^+^ T cells, which do not express any inhibitory receptors. (Fig. 6G).

## Discussion

Numerous preclinical studies and clinical trials have shown aldesleukin (rhIL-2) to be an effective antitumor therapy, driving durable responses in RCC and metastatic melanoma (1, 2). However, its use in the clinic has been limited by short half-life, severe VLS toxicity, and preferential activation of immune suppressive Treg cells. Multiple approaches have been pursued to improve the therapeutic index of IL-2, aiming to retain antitumor efficacy while minimizing toxicity and immune suppression (12, 13, 35). Most such efforts have focused on a “not-alpha” pharmacologic profile, to retain potency at target effector CD8^+^ T and NK cells with minimal or no induction of immunosuppressive Tregs and to avoid VLS.

Early strategies for increasing the therapeutic index of IL-2 involved random covalent single pegylation (36) or multiple reversible pegylation (37, 38) of lysine residues, seeking to increase half-life while modulating drug exposure with an aim to limit serious adverse sequelae. Although these approaches have shown varying degrees of antitumor efficacy in preclinical or early clinical testing (39, 40), they have not been completely successful in limiting adverse events (39, 41, 42). Other strategies have sought to modify receptor selectivity and widen the therapeutic index by (i) using selective IL-2/anti-IL-2 monoclonal antibody (mAb) complexes (9, 43, 44) or (ii) by generating IL-2 muteins that bias IL-2R chain engagement with decreased affinity for CD25 and conserved or increased potency for engagement of CD122. Although the first approach led to efficient activation of target cell types and generated remarkable antitumor responses in mouse models without causing VLS (9, 43, 44), it has nonetheless been very difficult to translate the strategy into a viable clinical treatment for patients with cancer, largely because of challenges to consistent formulation and lack of stability of the IL-2/antibody complexes *in vivo* (44, 45). The alternative approach for biased IL-2 stimulation via IL-2 muteins that favor IL-2R chain engagement with decreased affinity for CD25, and conserved or increased potency for engagement of CD122, has led to some success against solid murine tumors (46–48). Umaña and colleagues have reported on multiple not-alpha IL-2 fusions (IL-2v) that aim at localizing IL-2 at the target tumor site employing CEA (cergutuzumab amunaleukin; ref. 49) and FAP (simlukafusp alfa; ref. 50) engagement arms on the IgG construct. More recently, this same team showed that an IgG fusion that localizes IL-2v via engagement of PD-1 on T cells seems to result in a more durable effector phenotype that they dubbed “better effectors” (51). One issue with IL-2 variants seems to be development of immunogenicity and generation of antidrug antibodies (46). A different approach involved targeted engagement of memory CD8^+^ T cells by keeping IL-2Rα affinity intact while decreasing engagement of IL-2Rβ. An example is the IL-2 mutein BAY50-4798 (harboring the N88R mutation). It induced antidrug antibodies in 27% of treated patients after two cycles of treatment and showed low antitumor activity in patients with metastatic melanoma or RCC, malignancies that generally respond to high-dose IL-2 (52). After the discovery of CD4 Treg cells that express high levels of CD25 on the surface, this approach was resurrected by several groups to selectively target the activation and expansion of these cells for therapeutic intervention in autoimmune diseases (53, 54).

We leveraged our Expanded Genetic Alphabet platform to engineer a next-generation IL-2 Synthorin molecule for the treatment of tumors by site—specifically conjugating IL-2 with 30 kDa PEG to make it “not alpha”, with an improved PK and safety profile. Our NHP studies show that the half-life of SAR’245 is around 10 hours, much longer than that of aldesleukin in preclinical species or humans (10). Here, we have shown that the targeted, single-site pegylation increases exposure and leads to enhanced, durable peripheral CD8^+^ T and NK cell expansion in NHPs following i.v. dosing. Site-specific pegylation efficiently blocks the binding of SAR’245 to IL-2Rα, the receptor chain abundant not only on Treg cells, but also in type 2 innate lymphoid cells (IL-C-2) present in the vascular endothelium. Activation of type 2 IL-Cs has been suggested to induce eosinophilia and VLS associated with aldesleukin therapy (7). Our data also show minimal expansion of eosinophils. At the highest dose level (1 mg/kg) tested, one of two animals exhibited moderate expansion of eosinophils (Fig. 1C; Supplementary Fig. S3C). This differs from previous studies with aldesleukin in monkeys and humans, in which eosinophilia was observed from relatively low dose levels and lethality at 0.428 mg/kg (5, 6, 15, 16, 24). As aldesleukin was not included in our NHP studies, the safety and hematology data of aldesleukin was referred to the available information from publications. The lack of such observations with SAR’245 suggests that reducing binding to CD25 will lead to an increase in the therapeutic window by significantly reducing systemic toxicity.

Immune checkpoint blockade of PD-1/PD-L1 is recognized as a foundational approach in immuno-oncology, but complete and durable response rates remain low (55, 56). Although the checkpoint inhibition hypothesis is compelling for modification of the tumor microenvironment, such agents cannot elicit polyclonal expansion of antitumoral CD8^+^ Teff and NK cells, nor do they consistently modulate trafficking of immune cells into tumor sites or enhance expression of tumor antigens to improve antitumor immune responses. Therefore, we hypothesize that combining PD-1/PD-L1 blockade with SAR’245 would enhance the actions of checkpoint inhibitors with an agent that has the potential to increase the trafficking of immune cells into tumors and to stimulate expansion of intra-tumoral activated T-cells by promoting their proliferation and survival, as SAR’245 does. Preclinical mouse tumor model studies (Ma and colleagues; submitted for publication) with SAR’245 provide early proof of the mechanism for the viability of such a combination. To test the potential of SAR’245 in combination with immune checkpoint inhibitors in humans, we measured the effects of SAR’245 in combination with anti-PD-1 antibodies employing a MLR assay. In addition to demonstrating enhanced T-cell responses elicited by SAR’245 alone and with anti-PD-1, this experimental system showed that the combination did not induce the release of cytokines associated with CRS including IL-6, TNFα, and IL-8. Additionally, IL-5, which is associated with eosinophilia and VLS induced by high-dose IL-2, did not significantly change in response to SAR’245 treatment alone or in combination with pembrolizumab or nivolumab (8). These results further suggest a low risk of cytokine release from SAR’245 treatment as monotherapy and in combination with anti-PD-1 medicines in the clinical setting. Furthermore, different dosing schedules (Q1W–Q4W) used for testing SAR’245 in NHP demonstrated similar maximal PD responses, and the PD effect from the repeat doses reached similar levels of response relative to the first dose. Overall, our findings suggest that SAR’245 has broad flexibility when it comes to dosing regimens, which may prove useful if combined with immune-oncology therapies.

## Supplementary Material

Figure S1 Figure S1

Figure S2 Figure S2

Figure S3 Figure S3

Figure S4 Figure S4

Figure S5 Figure S5

Figure S6 Figure S6

Table S1 Table S1

Table S2 Table S2

Table S3 Table S3

Table S4 Table S4

Table S5 Table S5

Table S6 Table S6
