# Supplementary material for: A Precision Engineered Interleukin-2 for Bolstering CD8+ T- and NK-cell Activity without Eosinophilia and Vascular Leak Syndrome in Nonhuman Primates
Source: Cancer Res Commun. 2024 Oct 25;4(10):2799–814. doi: 10.1158/2767-9764.CRC-24-0278 (PMC11503527; doi:10.1158/2767-9764.CRC-24-0278)
Supplement: Figure S1 [file crc-24-0278_figure_s1_suppsf1.pdf]

Supplementary Figure S1

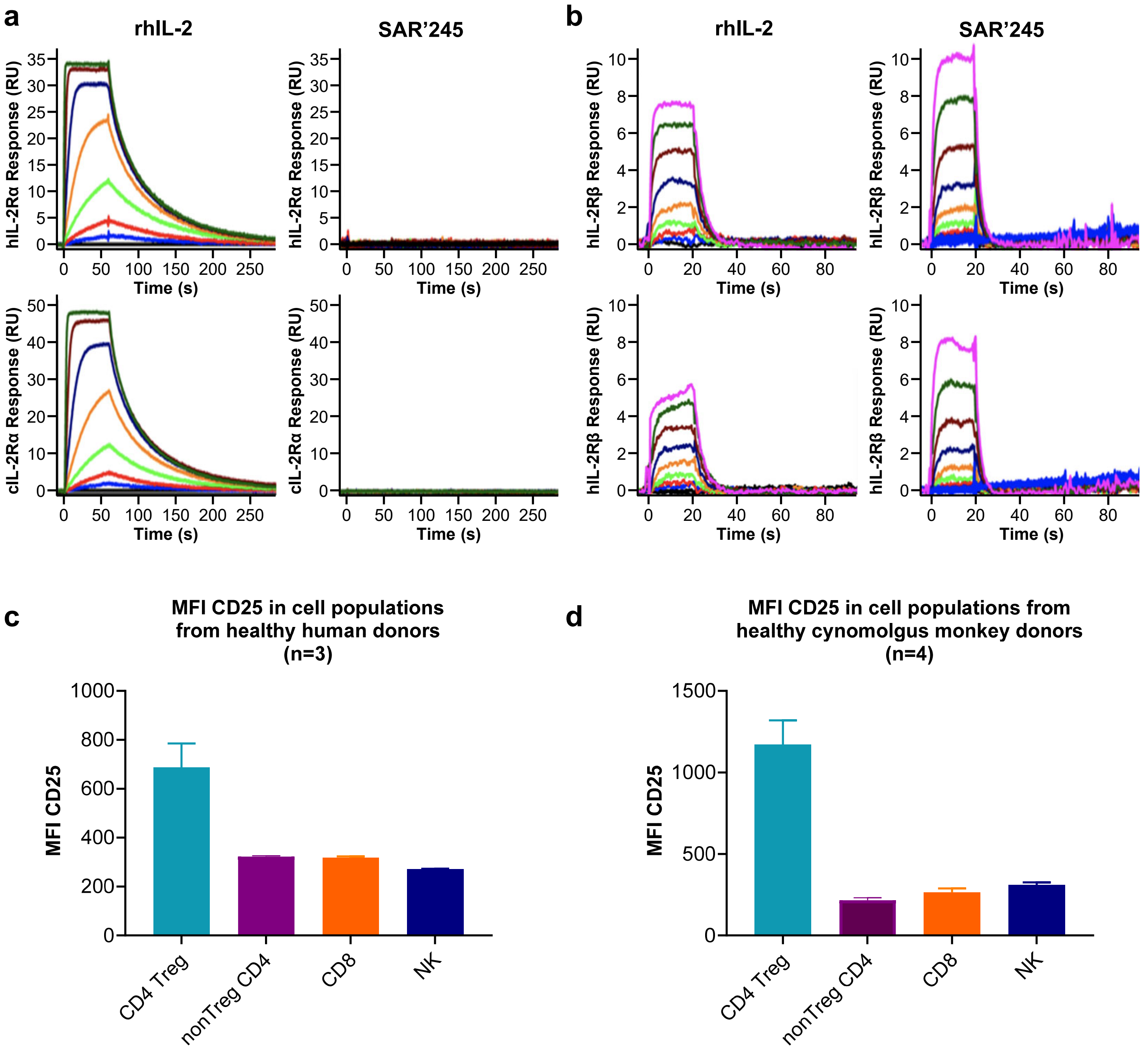

Supplementary Figure S1. Biochemical characterization of rhIL-2 and SAR’245 interactions with human and cynomolgus IL-2 receptor α and β extracellular domains using SPR (**a**,**b**). Representative binding profiles from human (top row) and cynomolgus (bottom row) IL-2Rα (**a**) and IL-2 Rβ (**b**); extracellular domains were immobilized on the surface of an SPR sensor and probed with two-fold serial dilutions of either rhIL-2 (left panels) or SAR’245 (right panels). rhIL-2 concentration was varied up to 1.65 μM and SAR’245 up to 3.3 μM. Color lines correspond to each test concentration. Test samples were injected for 60 s to measure association kinetics, followed by buffer only (wash) to measure dissociation kinetics. Response units (RU, Y-axis) are plotted versus time (s, X-axis). Panels **c** and **d** show MFI (median fluorescence intensity) of CD25 in CD4 Treg, CD8 and NK cells from healthy human and cynomolgus donors. Fresh PBMC cells were used for this analysis (n≥3).

cIL, cynomolgus interleukin; hIL, human interleukin; IL, interleukin; MFI, median fluorescence intensity; NK, natural killer; PBMC, peripheral blood mononuclear cells; rhIL-2, recombinant interleukin-2; RU, response units.
