# Supplementary material for: A Precision Engineered Interleukin-2 for Bolstering CD8+ T- and NK-cell Activity without Eosinophilia and Vascular Leak Syndrome in Nonhuman Primates
Source: Cancer Res Commun. 2024 Oct 25;4(10):2799–814. doi: 10.1158/2767-9764.CRC-24-0278 (PMC11503527; doi:10.1158/2767-9764.CRC-24-0278)
Supplement: Figure S2 [file crc-24-0278_figure_s2_suppsf2.pdf]

Supplementary Figure S2

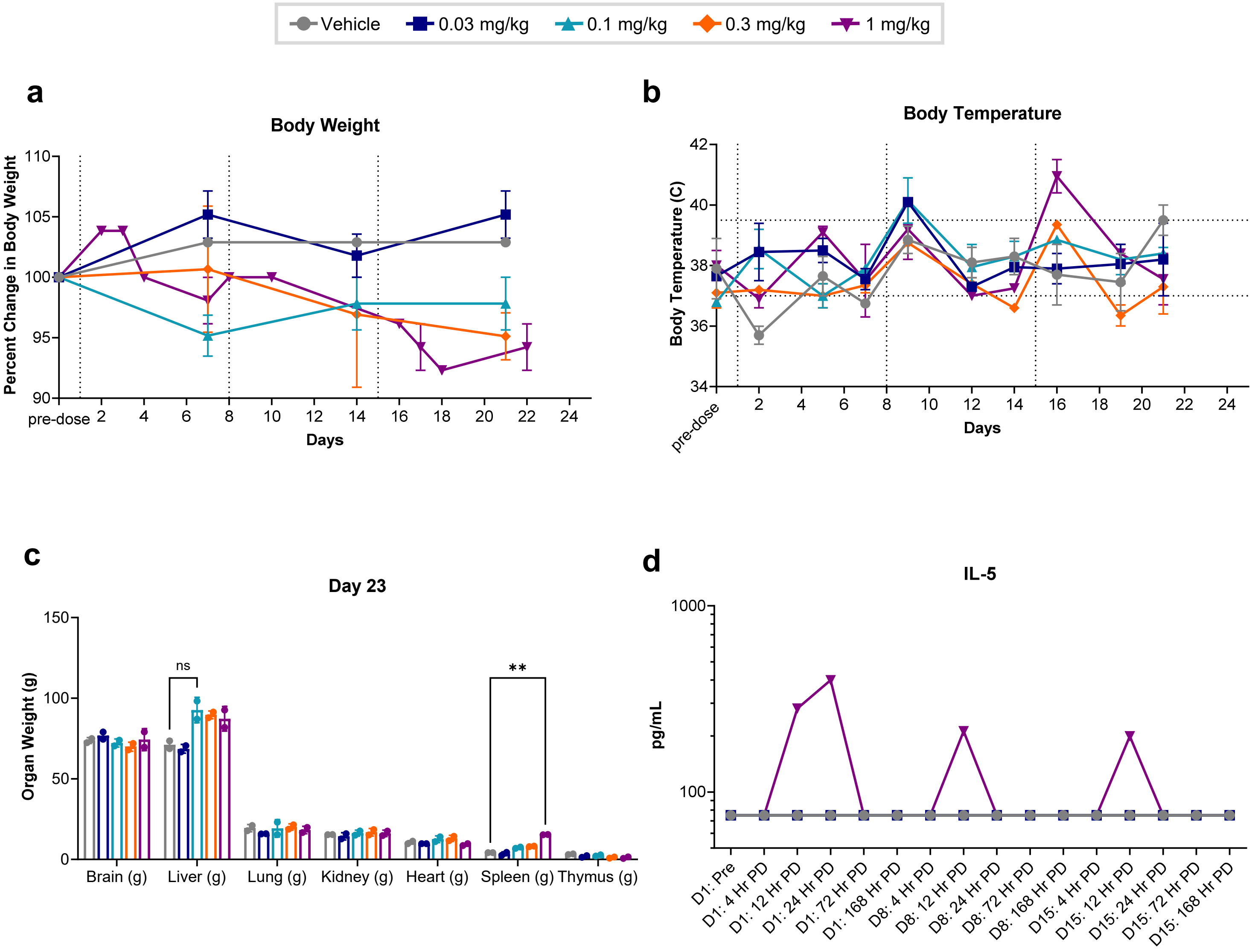

Supplementary Figure S2. Safety profile of SAR'245 in a dose-range finding study in cynomolgus monkeys. SAR'245 was given intravenously at 0.03, 0.1, 0.3, 1 mg/kg vs vehicle once a week for three dosing cycles (Days 1, 8 and 15). (**a**) Body weight, (**b**) body temperature, (**c**) organ weights, and (**d**) peripheral IL-5 reported at indicated time points throughout the study, or at end of study. (**a-c**) Mean ± SEM. N=2. (**d**) Each line represents an individual donor. N=2. Significance calculated by one-way ANOVA.
Hr, hour; Pre, predose; PD, postdose.
