# Supplementary material for: A Precision Engineered Interleukin-2 for Bolstering CD8+ T- and NK-cell Activity without Eosinophilia and Vascular Leak Syndrome in Nonhuman Primates
Source: Cancer Res Commun. 2024 Oct 25;4(10):2799–814. doi: 10.1158/2767-9764.CRC-24-0278 (PMC11503527; doi:10.1158/2767-9764.CRC-24-0278)
Supplement: Figure S3 [file crc-24-0278_figure_s3_suppsf3.pdf]

Supplementary Figure S3

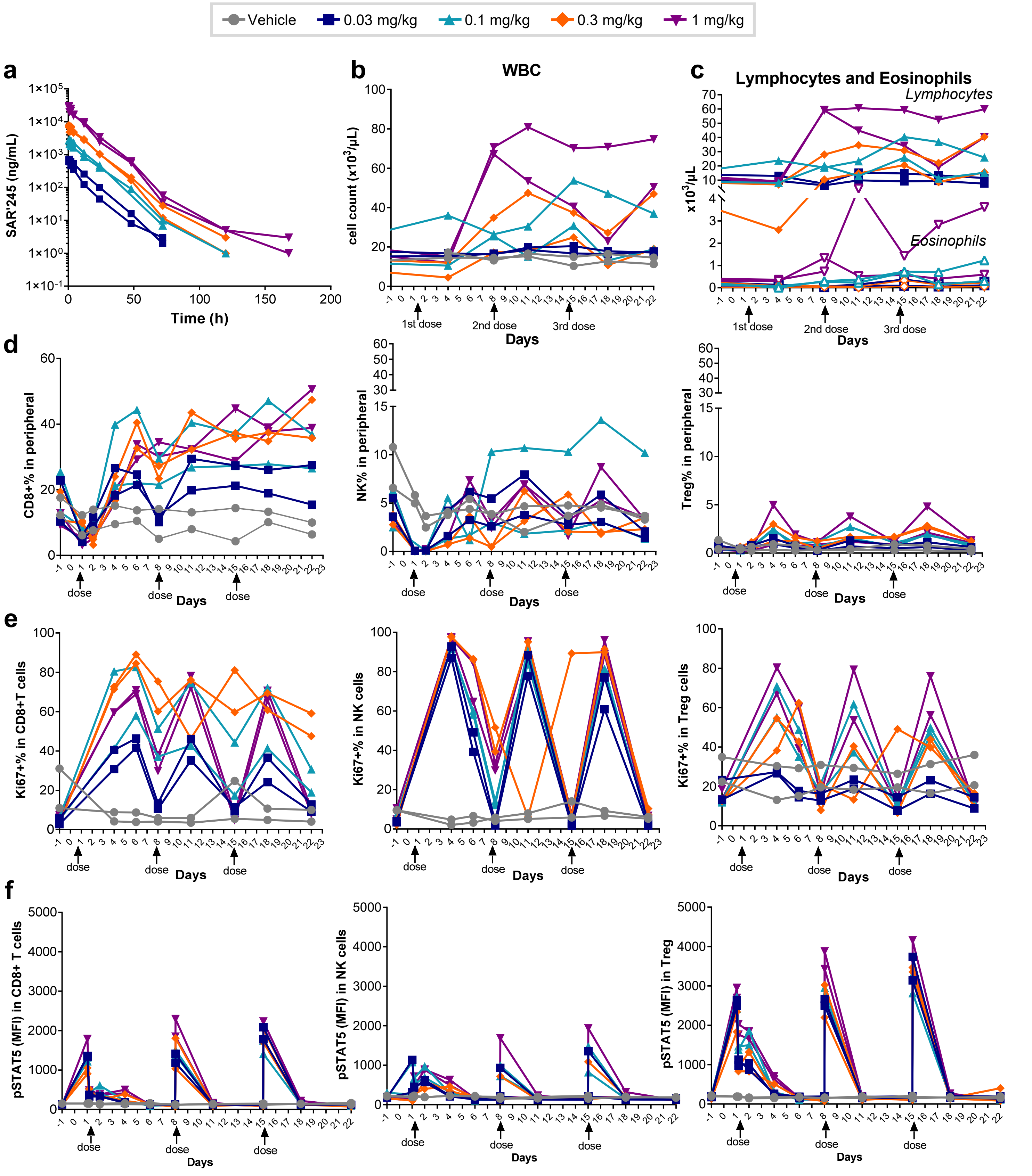

Supplementary Figure S3. Pharmacokinetic profile of SAR'245 in a dose-range finding study in cynomolgus monkeys. SAR'245 was given intravenously at 0.03, 0.1, 0.3, 1 mg/kg vs vehicle once a week for three dosing cycles (Days 1, 8 and 15). (**a**) SAR'245 concentration was measured from plasma samples collected from dosed treated animals following the first dose. Mean values are shown from two animals in each group; (**b**) Changes in WBC in response to repeat dosing with SAR'245 (c) Cell count changes in response to SAR'245 dosing for lymphocytes (•) and eosinophils (◦); (**d**) Percentage of CD8 T, NK and CD4 Treg cells in peripheral blood; (**e**) Percentage of Ki67 expression in CD8 T, NK and CD4 Treg cells in the peripheral blood; (**f**) MFI of pSTAT5 in CD8 T, NK and CD4 Treg cells in peripheral blood. N=2.

MFI, median fluorescence intensity; NK, natural killer; pSTAT5, phosphorylated signal transducer and activator of transcription 5.
