# Supplementary material for: A Precision Engineered Interleukin-2 for Bolstering CD8+ T- and NK-cell Activity without Eosinophilia and Vascular Leak Syndrome in Nonhuman Primates
Source: Cancer Res Commun. 2024 Oct 25;4(10):2799–814. doi: 10.1158/2767-9764.CRC-24-0278 (PMC11503527; doi:10.1158/2767-9764.CRC-24-0278)
Supplement: Figure S4 [file crc-24-0278_figure_s4_suppsf4.pdf]

Supplementary Figure S4

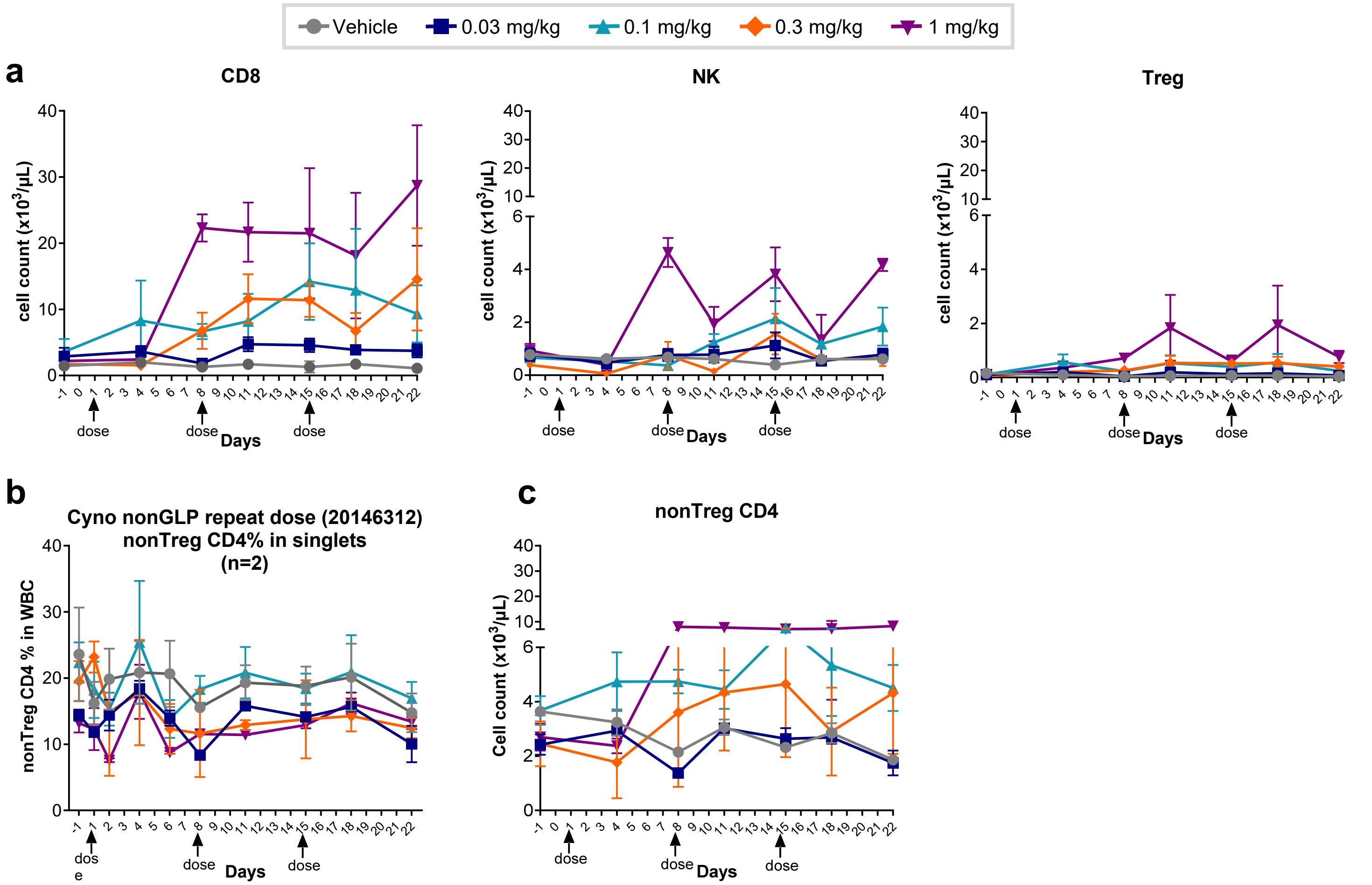

Supplementary Figure S4. Hematology and cell counts profile of SAR'245 in a dose-range finding study in cynomolgus monkeys. SAR'245 was given intravenously at 0.03, 0.1, 0.3, 1 mg/kg vs vehicle once a week for three dosing cycles (Days 1, 8 and 15). **(a)** CD8 T cell, NK, and Treg cell counts in the periphery. Cell counts are determined by WBC count x percentage of cells in WBC; **(b)** Percentage of CD4 T cells in WBC; and **(c)** CD4 T cell count in the periphery. Mean ± SEM. N=2.

Cyno, cynomolgus; GLP, good laboratory practice; WBC, white blood cell.
