# Supplementary material for: A Precision Engineered Interleukin-2 for Bolstering CD8+ T- and NK-cell Activity without Eosinophilia and Vascular Leak Syndrome in Nonhuman Primates
Source: Cancer Res Commun. 2024 Oct 25;4(10):2799–814. doi: 10.1158/2767-9764.CRC-24-0278 (PMC11503527; doi:10.1158/2767-9764.CRC-24-0278)
Supplement: Figure S5 [file crc-24-0278_figure_s5_suppsf5.pdf]

Supplementary Figure S5

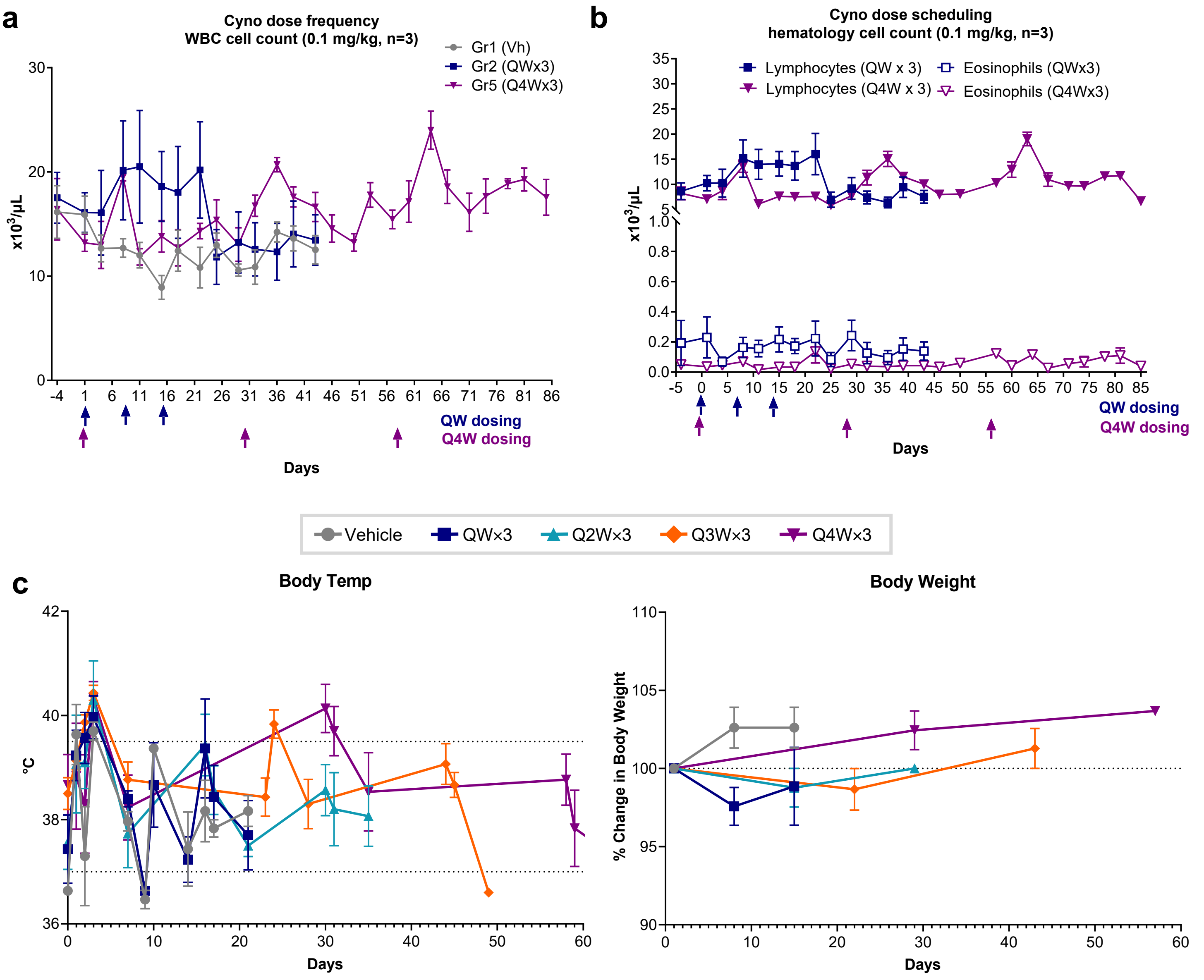

Supplementary Figure S5: Hematology profiles of SAR’245 in NHP dose scheduling study. SAR’245 was administered intravenously at 0.1mg/kg or with vehicle at QW, Q2W, Q3W or Q4W for three dosing cycles. Cell counts are shown as the mean+/- SEM, N=3 animals. **(a)** Changes in WBC from QW and Q4W groups in response to repeat dosing with SAR’245; **(b)** Changes of lymphocyte and eosinophil counts from QW and Q4W groups in response to repeat dosing of SAR’245; **(c)** Body temperature and body weight measurements over time.
Cyno, cynomolgus; WBC, white blood cell.
