# Supplementary material for: A Precision Engineered Interleukin-2 for Bolstering CD8+ T- and NK-cell Activity without Eosinophilia and Vascular Leak Syndrome in Nonhuman Primates
Source: Cancer Res Commun. 2024 Oct 25;4(10):2799–814. doi: 10.1158/2767-9764.CRC-24-0278 (PMC11503527; doi:10.1158/2767-9764.CRC-24-0278)
Supplement: Figure S6 [file crc-24-0278_figure_s6_suppsf6.pdf]

Supplementary Figure S6

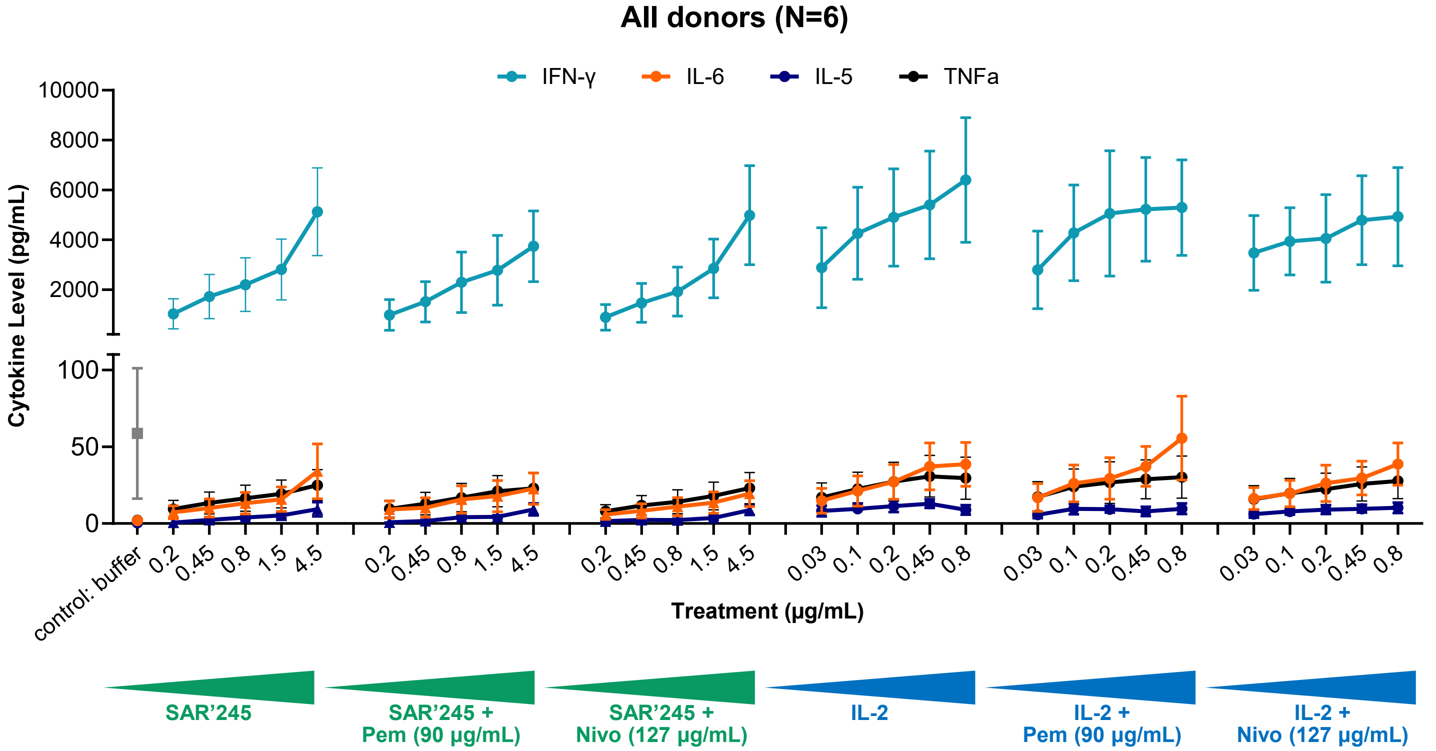

Supplementary Figure S6: SAR’245 did not elicit pan-cytokine release from human whole blood alone or in combination with anti-PD-1 antibodies. Human whole blood samples from 6 donors were independently treated for 24 h with varying concentrations of SAR’245 alone, rhIL-2 alone, or in combination with 90 µg/mL pembrolizumab or 127 µg/mL nivolumab. Cytokine release into culture supernatants was measured with an MSD 6-analyte panel (IFN-γ, TNF-α, IL-5, IL-6, IL-4, IL-8). TNF-α, IL-4, IL-8 are not shown. N=6, average ± SEM.

IFN-γ, interferon gamma; IL, interleukin; rhIL-2, recombinant interleukin-2; TNF-α, tumor necrosis factor alfa.
