# Supplementary material for: A Precision Engineered Interleukin-2 for Bolstering CD8+ T- and NK-cell Activity without Eosinophilia and Vascular Leak Syndrome in Nonhuman Primates
Source: Cancer Res Commun. 2024 Oct 25;4(10):2799–814. doi: 10.1158/2767-9764.CRC-24-0278 (PMC11503527; doi:10.1158/2767-9764.CRC-24-0278)
Supplement: Table S1 [file crc-24-0278_table_s1_suppst1.pdf]

Supplementary Table S1. List of compounds used in SPR Studies

Compound	Manufacturer	Cat# and lot#
rhIL-2	R&D Systems	#202-IL/CF, Lot AE6017121
SAR’245	Synthorx	Lot 008A, lyophilized
hIL2Ra His-tag	Sino Biological	Catalog #10165-H08H, Lot LC11DE2721
cIL2Ra His-tag	Sino Biological	Catalog #90265-C08H, Lot LC10SE1811
hIL2Rb His-tag	Sino Biological	Catalog #10696-H08B, Lot LC12MC1513
cIL2Rb His-tag	Creative BioMart	Catalog #IL2RB-619C, Lot 104886

cIL, cynomolgus interleukin; hIL, human interleukin; IL, interleukin; rhIL, recombinant human interleukin.
