# Supplementary material for: A Precision Engineered Interleukin-2 for Bolstering CD8+ T- and NK-cell Activity without Eosinophilia and Vascular Leak Syndrome in Nonhuman Primates
Source: Cancer Res Commun. 2024 Oct 25;4(10):2799–814. doi: 10.1158/2767-9764.CRC-24-0278 (PMC11503527; doi:10.1158/2767-9764.CRC-24-0278)
Supplement: Table S2 [file crc-24-0278_table_s2_suppst2.pdf]

Supplementary Table S2. Antibodies used in Phospho flow cytometry

4E-BP1 (T36/T45)	IkB-alpha (L35A5)	PKA RIIalpha (S99)
4E-BP1 (T37)	IKK alpha/beta (S176/180)	PKCa (T497)
4E-BP1 (T69)	IKKy	PLC (Y783)
Active Caspase-3	IRF-3 (S396)	PLC g2 (Y759)
Akt (S473)	IRF-4	PLC g1 (S1248)
AKT (T308)	IRF-7 (S477/S479)	PLK1 (T210)
AMPKbeta (S182)	IRS-1 (S616)	Rb (S780)
ATF-2 (T71)	IRS-1 (Y896)	Rb (S807/S811)
ATM (S1981)	Keratin 17 (S44)	RelB (S552)
b Catenin	Ki-67	S6 (S235/S236)
Bad (S112)	LAT (Y171)	S6 (S240)
Bcl-2 (S70)	LAT (Y226)	S6 Ribosomal (S240/244)
BLNK (Y84)	Lck (Y505)	SAMHD1 (T592)
Btk/Itk (Y223/Y180)	MAPKAPK-2 (T334)	SAPK/JNK T183/Y185)
BTK/ITK (Y551/Y511)	MARCKS (S167/170)	Sek1/mkk4 (S257)
c Cbl (Y700)	MCM2 (S139)	SHIP2 (Y1135)
c Cbl (Y774)	MEK1 (S298)	SHP2 (Y542)
CD247 (Y142)	MEK1/2 (S221)	SHP-2 (Y580)
CD79A (Y182)	MEK1/MEK2 (S218/S222)	SLP-76 (S376)
C-Fos	MNK (T197+202)	SLP-76 (Y128)
Chk1 (S345)	mTOR (S2448)	Smad2/3 (S465/S467/S423/S425)
c-Jun (S73)	NDRG1 (T346)	Src (Y418)
Cleaved PARP (D214)	NF kB p65 (S529)	STAT1 (Y701)
CREB/ATF1 (S133/S63)	NF-kB (S536)	STAT1 (S727)

CrkL (Y207)	NPM (S4)	STAT3 (Y705)
DRP1 (S616)	p120 Catenin (S288)	STAT3 (S727)
eIF4E (S209)	p120 Catenin (S879)	STAT4
ELK	p120 Catenin (T310)	STAT5
Elk-1 (S383)	p120 Catenin (T916)	STAT6
ERK ½ (T202/Y204)	p38 MAPk (T180/Y182)	Syk (Y348)
Ezrin (Y353)	p44/42 Erk1/2 (T202/Y204)	Syk (Y525/526)
FAK (S910)	p53 (K382)	TBK1/NAK (S172)
FAK (Y576)	p53 (S15)	TCTP (S46)
GSK3B (S9)	p53 (S37)	Vimentin (S83)
H2AX (S139)	p70 s6 (T389)	WIP (S488)
Helios	p70 s6 (T421+S4)	Yk2 (Y402)
Histone H3 (S10)	p90RSK (S380)	ZAP70 (Y292)
Histone H3 (S28)	pChk2 (T68)	ZAP70/SYK (Y319/Y352)
HS1 (Y397)	PDPK1 (S241)	
HSP27 (S82)	Phosphotyrosine	
