# Supplementary material for: A Precision Engineered Interleukin-2 for Bolstering CD8+ T- and NK-cell Activity without Eosinophilia and Vascular Leak Syndrome in Nonhuman Primates
Source: Cancer Res Commun. 2024 Oct 25;4(10):2799–814. doi: 10.1158/2767-9764.CRC-24-0278 (PMC11503527; doi:10.1158/2767-9764.CRC-24-0278)
Supplement: Table S3 [file crc-24-0278_table_s3_suppst3.pdf]

Supplementary Table S3. hIL-2 and SAR'245 engagement with IL-2Rα and IL-2Rβ chains measured via surface plasmon resonance (SPR). IL-2, interleukin-2;IL-2Rα, interleukin 2 receptor alfa chain; IL-2Rβ, interleukin 2 receptor beta chain; ND, not detectable.

Species	Human IL-2 K_D (nM)		SAR'245 K_D (nM)	
	IL-2 Rα	IL-2 Rβ	IL-2 Rα	IL-2 Rβ
Human	1.71	360	ND	1,400
Monkey	2.90	270	ND	1,200
