# Supplementary material for: A Precision Engineered Interleukin-2 for Bolstering CD8+ T- and NK-cell Activity without Eosinophilia and Vascular Leak Syndrome in Nonhuman Primates
Source: Cancer Res Commun. 2024 Oct 25;4(10):2799–814. doi: 10.1158/2767-9764.CRC-24-0278 (PMC11503527; doi:10.1158/2767-9764.CRC-24-0278)
Supplement: Table S4 [file crc-24-0278_table_s4_suppst4.pdf]

Supplementary Table S4. Potency of rhIL-2 and SAR'245 as measured by phosphorylation of STAT5 in primary Treg, CD8$^+$ T and NK cells from human and cynomolgus monkey. rhIL-2, recombinant human interleukin 2; NK, natural killer; STAT5, signal transducer and activator of transcription 5A; Treg, regulatory CD4$^+$ T cell.

Species	Human IL-2 EC_{50} (pSTAT5) (ng/mL)			SAR'245 EC_{50} (pSTAT5) (ng/mL)		
	CD4$^+$ Treg	CD8$^+$ T	NK	CD4$^+$ Treg	CD8$^+$ T	NK
Human	0.027 ± 0.005	12.4 ± 1.29	2.88 ± 1.63	114 ± 20.3	224 ± 25.3	54.7 ± 6.55
Monkey	0.047 ± 0.005	8.01 ± 0.34	3.28 ± 0.78	55.5 ± 3.15	98.6 ± 6.86	31.5 ± 9.87
