# Supplementary material for: A Precision Engineered Interleukin-2 for Bolstering CD8+ T- and NK-cell Activity without Eosinophilia and Vascular Leak Syndrome in Nonhuman Primates
Source: Cancer Res Commun. 2024 Oct 25;4(10):2799–814. doi: 10.1158/2767-9764.CRC-24-0278 (PMC11503527; doi:10.1158/2767-9764.CRC-24-0278)
Supplement: Table S5 [file crc-24-0278_table_s5_suppst5.pdf]

Supplementary Table S5. Mean plasma PK parameters of SAR'245 following the first IV bolus dose administration to naïve cynomolgus monkeys. $AUC_{0\text{-}168h}$, area under the concentration time curve of 0 h to 168 h; CL, clearance; C_{max}, maximum drug concentration; IV, intravenous; PK, pharmacokinetic; $T_{½}$, half-life; T_{max}, time to peak drug concentration; Vd, volume of distribution.

**Parameter**	**Units**	**SAR'245 Dose (mg/kg)**			
		**0.03**	**0.1**	**0.3**	**1**
**T_{max}**	h	0.75 ± 0.25	0.75 ± 0.25	0.5	0.5
**C_{max}**	µg/mL	0.674 ± 0.045	2.81 ± 0.085	7.36 ± 0.42	29.0 ± 0.755
**$AUC_{0\text{-}168h}$**	h•µg/mL	7.60 ± 1.91	39.5 ± 6.1	97.6 ± 3.065	316 ± 12.83
**$t_{1/2}$**	h	9.81 ± 1.335	9.35 ± 1.175	10.9 ± 1.82	11.2 ± 0.36
**CL**	mL/h/kg	4.19 ± 1.05	2.59 ± 0.405	3.08 ± 0.095	3.17 ± 0.125
**V_d**	mL/kg	61.3 ± 22.88	35.7 ± 9.84	48.1 ± 6.575	51.0 ± 3.725
