# Supplementary material for: A Precision Engineered Interleukin-2 for Bolstering CD8+ T- and NK-cell Activity without Eosinophilia and Vascular Leak Syndrome in Nonhuman Primates
Source: Cancer Res Commun. 2024 Oct 25;4(10):2799–814. doi: 10.1158/2767-9764.CRC-24-0278 (PMC11503527; doi:10.1158/2767-9764.CRC-24-0278)
Supplement: Table S6 [file crc-24-0278_table_s6_suppst6.pdf]

Supplementary Table S6. Mean plasma PK parameters of SAR'245 following the first and third IV doses of 0.1mg/kg to cynomolgus monkeys on various multiple dosing schedules. $AUC_{0\text{-}168h}$; area under the concentration time curve of 0 h to 168 h; CL, clearance; Cmax, maximum drug concentration; IV, intravenous; PK, pharmacokinetics; $T_{½}$, half-life; T_{max}, time to peak drug concentration; Vss, volume in steady state.

Parameter	Units	SAR'245 Dose Schedule			
		QW	Q2W	Q3W	Q4W
**Post First Dose**					
T_{max}	h	0.5	0.5	0.5	0.5
C_{max}	ng/mL	2,740 ± 255.43	3,190 ± 202.98	3,130 ± 187.71	2,890 ± 274.29
$AUC_{0\text{-}168h}$	h xng/mL	35,700 ± 2571.86	46,300 ± 1540.92	39,900 ± 4656.30	42,300 ± 1334.58
$t_{1/2}$	h	8.63 ± 0.30	7.88 ± 0.25	8.95 ± 0.48	8.94 ± 0.59
**CL**	mL/h/kg	2.83 ± 0.21	2.16 ± 0.07	2.57 ± 0.30	2.37 ± 0.08
**Vss**	mL/kg	31.3 ± 2.23	26.2 ± 0.47	26.3 ± 1.25	31.7 ± 2.54
**Post Third Dose** [a]					
T_{max}	h	0.5	1.25 ± 0.61	1.5 ± 0.50	0.5
C_{max}	ng/mL	2,390 ± 167.38	3,710 ± 289.86	3,870 ± 327.43	2,640 ± 140.75
$AUC_{0\text{-}168h}$	h x ng/mL	23,200 ± 1877.94	49,700 ± 1428.87	46,500 ± 4585.61	33,400 ± 3254.40
$t_{1/2}$	h	9.94 ± 0.05	8.39 ± 0.37	8.58 ± 0.78	9.59 ± 0.54
**CL**	mL/h/kg	4.36 ± 0.35	2.02 ± 0.05	2.08 ± 0.23	3.05 ± 0.32
**Vss**	mL/kg	31.8 ± 3.35	21.7 ± 0.45	21.1 ± 0.24	30.3 ± 2.05
